# Supplementary material for: Genome sequencing unveils a regulatory landscape of platelet reactivity
Source: Nat Commun. 2021 Jun 15;12:3626. doi: 10.1038/s41467-021-23470-9 (PMC8206369; doi:10.1038/s41467-021-23470-9)

Supplementary Table 1. Platelet aggregation results were harmonized across the cohorts. * indicates Maximum aggregation to ADP/Epinephrine and ** indicates Lag time to Collagen.

Phenotype	Agonist	Framingham	GeneSTAR	Amish	Sample size
adp_low1	ADP	* 1μM	* 2μM	* 2μM	3140
adp_low2	ADP	* 3μM	* 2μM	* 2μM	3229
adp_low3	ADP	Threshold dose for >50% aggregation	* 2μM	* 2μM	3014
adp_high1	ADP	* 5μM	* 10μM	* 5μM	2799
adp_high2	ADP	* 5μM	* 10μM	* 10μM	2799
adp_high3	ADP	* 10μM	* 10μM	* 10μM	1967
adp_high4	ADP	Threshold dose for >50% aggregation	* 10μM	* 10μM	3147
epi_low1	Epinephrine	* 0.5μM	* 2μM	* 10μM	2962
epi_low2	Epinephrine	* 1μM	* 2μM	* 10μM	3027
epi_low3	Epinephrine	* 3μM	* 2μM	* 10μM	2486
epi_low4	Epinephrine	* 3μM	* 10μM	* 10μM	2488
epi_low5	Epinephrine	Threshold dose for >50% aggregation	* 2μM	* 10μM	3152
epi_high1	Epinephrine	* 5μM	* 10μM	* 10μM	2098
epi_high2	Epinephrine	* 10μM	* 10μM	* 10μM	2141
epi_high3	Epinephrine	Threshold dose for >50% aggregation	* 10μM	* 10μM	3154
col_low1	Collagen	** 190 μg/mL	** 1μg/mL	** 1μg/mL	3354
col_low2	Collagen	** 190 μg/mL	** 2μg/mL	** 2μg/mL	3364
col_high1	Collagen	** 190 μg/mL	** 5μg/mL	** 5μg/mL	3361
col_high2	Collagen	** 190 μg/mL	** 10μg/mL	** 10μg/mL	3352

Supplementary Table 2. Demographics for participants included in genome-wide association analyses. Values given are % or mean ± 1 SD. FHS=Framingham Heart Study, Amish=Old Order Amish Study, GS=GeneSTAR, EA=European American, AA=African American.

Characteristic	FHS n=1,981	Amish n=235	GS EA n=909	GS AA n=730
Male, %	47.15	48.94	44.11	36.71
Diabetes, %	6.3	0.43	5.19	11.96
Hypertension, %	35.34	5.96	24.67	39.25
Smoking, %	17.47	8.94	20.59	31.85
Cardiovascular disease, %	6.11	2.55	1.1	1.78
Aspirin response, %	15.49	0	0	0
Age, years	55.76 ± 9.2	46.73 ± 13.6	44.5 ± 13.2	43.4 ± 12.4
Body mass index, kg/m2	27.52 ± 4.9	26.9 ± 4.4	28.7 ± 6.4	31.8 ± 8.1
LDL cholesterol, mg/dl	127.4 ± 32.8	144.6 ± 44.8	124.8 ± 37.4	120.6 ± 38.9
Fibrinogen, mg/dl	308.0 ± 56.2	281.9 ± 57.8	374.3 ± 113	417.3 ± 122
Maximal aggregation to low ADP doses (%)	19.0 ± 21.5 (1uM)<br>67.8 ± 24.7(3uM)	41.5 ± 22.5 (2uM)	44.6 ± 26.2 (2uM)	41.8 ± 28.6 (2uM)
Maximal aggregation to high ADP doses (%)	77.4 ± 18.9 (5uM)<br>78.0 ± 19.5 (10uM)	61.5 ± 18.4 (5uM)<br>67.5 ± 13.5 (10uM)	79.4 ± 13.3 (10uM)	77.0 ± 17.3 (10uM)
Threshold concentration (EC50) for 50% response to ADP (uM)	3.27 ± 1.5	NA	NA	NA
Maximal aggregation to low epinephrine doses (%)	51.2 ± 31.0 (0.5uM)<br>58.2 ± 31.1 (1uM)<br>66.0 ± 28.4 (3uM)	60.2 ± 27.1 (10uM)	56.1 ± 33.2 (2uM)	51.6 ± 36.0 (2uM)
Maximal aggregation to high epinephrine doses (%)	48.1 ± 28.6 (5uM)<br>34.8 ± 24.5 (10uM)	60.2 ± 27.1 (10uM)	71.8 ± 27.2 (10uM)	63.5 ± 34.1 (10uM)
Threshold concentration (EC50) for 50% response to epinephrine (uM)	1.92 ± 2.9	NA	NA	NA
Lag time to low collagen doses (seconds)	87.0 ± 25.2 (190ug/ml)	56.8 ± 24.9 (1ug/ml)<br>47.9 ± 17.5 (2ug/ml)	147.8 ± 87.6 (1ug/ml)<br>99.9 ± 57.5 (2ug/ml)	158.1 ± 90.4 (1ug/ml)<br>111.5 ± 67.3 (2ug/ml)
Lag time to high collagen doses (seconds)	87.0 ± 25.2 (190ug/ml)	37.9 ± 12.1 (5ug/ml)<br>32.6 ± 10.5 (10ug/ml)	68.8 ± 32.1 (5ug/ml)<br>60.5 ± 23.3 (10ug/ml)	77.4 ± 40.8 (5ug/ml)<br>64.1 ± 26.4 (10ug/ml)

Supplementary Table 3 Discovery and Replication results of the variants that drove the gene-based signals. The variants were identified through leave-one-out analysis. Replication was performed as a meta-analysis in order of FHS, GeneSTAR European Americans, GeneSTAR African Americans and OOA for direction of effect. P-values are from a two-sided score test with no adjustment for discovery and one sided Z test no adjustment for replication.

				DISCOVERY					REPLICATION			
Gene	snpID hg38	ref/alt	Phenotype	N	MAF	beta	se	p-value	Direction	N	Zscore	p-value
SVEP1	9:110549951	G/C	adp_low1	3140	0.029	0.338	0.075	5.84E-06	++?+	1833	2.662	0.004
BCO1	16:81290348	G/C	epi_low1	2962	0.007	0.719	0.147	1.05E-06	--??	1195	-0.575	0.717
IDH3A	15:78161708	T/A	col_high2	3352	0.006	0.742	0.153	1.20E-06	--??	1510	-1.012	0.844

Supplementary Table 4: Platelet aggregation results in the Caerphilly Prospective Study. Top, Lead variants and RGS18 genome-wide significant variants. Bottom, Variants driving gene-based SKAT. P-values are from a one-sided Z test with no adjustment for multple-correction.

						Trait: ADP (0.725uM)				Trait: Collagen (42.7 ug/mL)			
snpID hg38	rsID	Ref / Alt	minor allele	MAF	Rsq	N	beta	se	p-value	N	beta	se	p-value
1:156899922	rs12041331	G/A	ALT	0.08	0.992	1177	-0.304	0.055	1.62E-08	811	-0.688	0.145	9.75E-07
1:192194880	rs1175170	G/C	ALT	0.487	0.987	1177	0.087	0.029	1.09E-03	811	0.203	0.075	3.52E-03
1:20567949	rs12137738	A/T	ALT	0.108	0.961	1177	-0.014	0.046	6.20E-01	811	-0.02	0.126	5.64E-01
1:67128641	rs142001088	C/T	ALT	0.023	0.887	1177	-0.04	0.100	6.56E-01	811	-0.5	0.259	9.73E-02
10:111139289	rs7097060	T/A	ALT	0.188	0.988	1177	-0.046	0.038	1.11E-01	811	-0.074	0.100	2.31E-01
11:92185065	rs183146849	A/T	ALT	0.019	0.863	1177	0.094	0.110	8.02E-01	811	-0.444	0.297	9.33E-01
12:132589485	rs140148392	G/A	ALT	0.014	0.934	1177	0.144	0.123	8.79E-01	811	0.506	0.322	9.42E-01
13:96912429	rs61974290	A/G	ALT	0.067	0.963	1177	0.011	0.057	5.79E-01	811	-0.054	0.151	6.40E-01
5:19109993	rs112157462	T/C	ALT	0.028	0.892	1177	0.063	0.092	7.53E-01	811	-0.259	0.260	1.59E-01
6:121921871	rs58250884	A/G	ALT	0.052	0.997	1177	-0.024	0.065	3.58E-01	811	0.126	0.180	7.59E-01
1:192139135	rs12070423	A/G	ALT	0.496	0.983	1177	0.093	0.029	6.08E-04	811	0.239	0.076	8.29E-04
1:192140500	rs10801100	T/A	ALT	0.496	0.985	1177	0.093	0.029	5.95E-04	811	0.239	0.076	7.93E-04
1:192141301	rs7546592	A/C	ALT	0.496	0.985	1177	0.093	0.029	5.91E-04	811	0.239	0.076	7.96E-04
1:192144114	rs10801102	G/A	ALT	0.479	0.986	1177	0.095	0.029	4.82E-04	811	0.221	0.076	1.81E-03
1:192148472	rs6687273	C/T	ALT	0.495	0.994	1177	0.093	0.029	6.08E-04	811	0.233	0.075	9.91E-04
1:192154581	rs7526348	A/G	ALT	0.495	0.998	1177	0.093	0.029	5.68E-04	811	0.235	0.075	9.03E-04
1:192168015	rs10754003	T/A	ALT	0.493	0.993	1177	0.096	0.029	3.85E-04	811	0.235	0.075	8.86E-04
1:192174246	rs12117018	T/G	ALT	0.48	0.992	1177	0.092	0.029	5.95E-04	811	0.205	0.075	3.17E-03
1:192182044	rs1937235	A/C	ALT	0.487	0.989	1177	0.088	0.029	1.05E-03	811	0.203	0.075	3.54E-03
1:192183215	rs10921107	C/A	ALT	0.473	0.989	1177	0.088	0.029	1.03E-03	811	0.183	0.075	7.42E-03
1:192186870	rs2247567	T/C	ALT	0.473	0.989	1177	0.088	0.029	1.05E-03	811	0.183	0.075	7.42E-03
1:192186916	rs2247566	G/A	ALT	0.471	0.988	1177	0.088	0.029	1.00E-03	811	0.18	0.076	8.67E-03
1:192193726	rs1175168	T/C	ALT	0.472	0.988	1177	0.088	0.029	1.06E-03	811	0.184	0.075	7.34E-03
1:192193819	rs1175169	T/C	ALT	0.487	0.988	1177	0.088	0.029	1.07E-03	811	0.204	0.075	3.40E-03
1:192194880	rs1175170	G/C	ALT	0.487	0.987	1177	0.087	0.029	1.09E-03	811	0.203	0.075	3.52E-03
1:192195284	rs1175171	T/C	ALT	0.487	0.987	1177	0.087	0.029	1.09E-03	811	0.203	0.075	3.54E-03
1:192206244	rs12037701	C/T	REF	0.49	0.982	1177	-0.087	0.028	1.08E-03	811	-0.15	0.075	2.25E-02
snpID hg38	rsID	REF	minor allele	MAF	Rsq	N	beta	Se	p-value	Gene		Trait	
9:110549951	rs61751937	G/C	ALT	0.018	0.999	1177	0.251	0.104	7.98E-03	SVEP1		ADP	
16:81290348	rs143238312	G/C	ALT	0.013	0.989	1177	0.245	0.128	2.74E-02	BCO1		ADP	
16:81290348	rs143238312	G/C	ALT	0.013	0.989	1183	0.365	0.163	1.27E-02	BCO1		Thrombin	

Supplementary Table 5a. Comparison of results from previous Hapmap study and current TOPMed study for the five previously identified variants not replicated in current study. P-values are from a two-sided score test with no adjustment for multiple testing for the Mega analysis and two-sided Z test for other analyses. *: percentage (in %) of TOPMed sample size overlapped in corresponding Hapmap sample size. For example, FHS has N=1350 for adp_high4 in TOPMed and 1258 of them are in Hapmap FHS (N=2372), 1258/2372=53%. ** Hapmap Meta used sample size weighted approach that only reports Z statistic instead of beta and se.

chr:pos (hg38)	rsID	ref/alt	Phenotype (see Table S1)	Analysis	N (%)*	MAF	beta	se	p-value
11:10647681	rs7940646	T/C	adp_high2	TOPMed Mega	2799	0.273	-0.122	0.031	9.90E-05
				TOPMed Meta	2799	0.27	-3.763**	-	1.68E-04
				TOPMed Amish	234 (0%)	0.476	-0.071	0.1	4.76E-01
				TOPMed FHS	1002 (52%)	0.312	-0.129	0.051	1.05E-02
				TOPMed GS_EA	887 (72%)	0.309	-0.148	0.053	5.19E-03
				TOPMed GS_AA	676 (0%)	0.083	-0.097	0.105	3.57E-01
				Hapmap Meta	3030	0.314	-5.61**	-	2.03E-08
				Hapmap FHS	1803	0.312	-0.03	0.007	1.58E-05
				Hapmap GS_EA	1227	0.318	-2.145	0.582	2.45E-04
7:155968169	rs2363910	T/G	adp_high4	TOPMed Mega	3147	0.09	-0.161	0.046	4.59E-04
				TOPMed Meta	3147	0.09	-3.375**	-	7.39E-04
				TOPMed Amish	234 (0%)	0.017	-0.077	0.359	8.31E-01
				TOPMed FHS	1350 (53%)	0.077	-0.147	0.071	3.92E-02
				TOPMed GS_EA	887 (72%)	0.097	-0.228	0.084	6.67E-03
				TOPMed GS_AA	676 (0%)	0.131	-0.099	0.088	2.57E-01
				Hapmap Meta	3482	0.078	-5.47**	-	4.50E-08
				Hapmap FHS	2372	0.071	-0.043	0.013	6.10E-04
				Hapmap GS_EA	1110	0.092	-4.518	0.955	2.59E-06
10:63306426	rs10761741	G/T	epi_low5	TOPMed Mega	3152	0.402	0.106	0.026	5.61E-05
				TOPMed Meta	3152	0.402	4.021**	-	5.80E-05
				TOPMed Amish	235 (0%)	0.462	-0.089	0.098	3.65E-01
				TOPMed FHS	1343 (53%)	0.41	0.157	0.038	3.62E-05
				TOPMed GS_EA	896 (72%)	0.425	0.101	0.051	4.63E-02
				TOPMed GS_AA	678 (0%)	0.335	0.067	0.061	2.72E-01
				Hapmap Meta	3602	0.417	5.653**	-	1.57E-08
				Hapmap FHS	2364	0.415	0.082	0.017	1.47E-06
				Hapmap GS_EA	1238	0.422	4.053	1.31	2.04E-03
7:106724153	rs342286	A/G	epi_low5	TOPMed Mega	3152	0.359	-0.137	0.027	4.04E-07
				TOPMed Meta	3152	0.359	-4.351**	-	1.35E-05
				TOPMed Amish	235 (0%)	0.262	-0.126	0.11	2.52E-01
				TOPMed FHS	1343 (53)	0.439	-0.141	0.037	1.55E-04
				TOPMed GS_EA	896 (72%)	0.449	-0.163	0.049	8.15E-04
				TOPMed GS_AA	678 (0%)	0.114	0.041	0.09	6.43E-01
				Hapmap Meta	3602	0.435	-5.925**	-	3.12E-09
				Hapmap FHS	2364	0.441	-0.086	0.017	4.15E-07
				Hapmap GS_EA	1238	0.422	-4.472	1.388	1.32E-03
19:55014977	rs1671152	T/G	col_low2	TOPMed Mega	3364	0.188	-0.161	0.033	1.04E-06
				TOPMed Meta	3364	0.188	-5.283**	-	1.27E-07
				TOPMed Amish	234 (0%)	0.252	-0.048	0.111	6.66E-01
				TOPMed FHS	1551 (51)	0.146	-0.301	0.052	6.27E-09
				TOPMed GS_EA	899 (73%)	0.152	-0.119	0.069	8.40E-02
				TOPMed GS_AA	680 (0%)	0.309	-0.047	0.063	4.62E-01

chr:pos (hg38)	rsID	ref/alt	Phenotype (see Table S1)	Analysis	N (%)*	MAF	beta	se	p-value
				Hapmap Meta	3472	0.148	-7.238**	-	4.57E-13
				Hapmap FHS	2310	0.142	-0.033	0.004	9.11E-14
				Hapmap GS_EA	1162	0.159	-0.017	0.008	3.71E-02

Supplementary Table 5b. Comparison of results from previous study and current study for RGS18 variant (rs1175170). P-values are from a two-sided score test with no adjustment for multiple testing for the Mega analysis and two-sided Z test for other analyses. *: percentage (in %) of TOPMed sample size overlapped in corresponding Hapmap sample size. For example, FHS has N=1350 for adp_high4 in TOPMed and 1258 of them are in Hapmap FHS (N=2372), 1258/2372=53%. ** Hapmap Meta used sample size weighted approach that only reports Z statistic instead of beta and se.

chr:pos (hg38)	rsID	ref/alt	Phenotype (see Table S1)	Analysis	N (%)*	MAF	beta	se	p-value
1:192194880	rs1175170	G/C	epi_low5	TOPMed Mega	3152	0.446	0.155	0.026	1.96E-09
				TOPMed Meta	3152	0.44	6.002**	-	1.95E-09
				TOPMed Amish	235 (0%)	0.394	0.201	0.098	3.92E-02
				TOPMed FHS	1343 (53%)	0.478	0.161	0.037	1.47E-05
				TOPMed GS_EA	896 (72%)	0.488	0.101	0.049	4.01E-02
				TOPMed GS_AA	678 (0%)	0.316	0.2	0.061	1.08E-03
				Hapmap Meta	3602	0.493	4.365**	-	1.27E-05
				Hapmap FHS	2364	0.485	0.065	0.017	1.36E-04
				Hapmap GS_EA	1238	0.492	3.357	1.494	2.49E-02

Supplementary Table 6: Co-localization between all transcripts having a platelet eQTL p-value <0.0031(0.05/16) within +/- 20KB of GWAS locus peak. Meaningful co-localization was noted between PEAR1 and RGS18 for Chr1:156899922 and Chr1:192194880 loci, respectively. Posterior probabilities for the H4 hypotheses from the Bayesian model is presented for the target gene.

Locus	Phenotype (see Table S1)	Co-localization by gene (posterior probability in %)
1:20567949	epi_low3	NBPF3 (9.54%)
1:67128641	col_high1	SLC35D1 (0.629%)
1:156899922	adp_low1	LMNA (2.64%), **PEAR1 (99.6%),**
1:156899922	col_high1	LMNA (2.64%), **PEAR1 (99.6%)**
1:156899922	epi_low1	LMNA (2.64%), **PEAR1 (99.6%)**
1:192194880	adp_high1	**RGS18 (69.7%)**
1:192194880	epi_low5	**RGS18 (69.0%)**
5:19109993	col_high1	-
6:121921871	epi_low2	-
9:28873884	epi_low2	-
10:75490891	col_high2	-
10:111139289	epi_high3	ADRA2A (34.5%)
11:92185065	adp_high2	-
12:132589485	col_high2	ANKLE2 (0.0818%), FBRSL1 (0.115%)
13:96912429	epi_high1	-
17:21960955	epi_low5	-
17:21960955	adp_low3	-
17:16451482	col_low2	LLRC75A-AS1 (1.67%)
18:29059923	col_low2	-
20:50142397	col_high2	BCAS4 (0.369%)

Supplementary Table 7: The BioVU Biobank and UKBB PheWAS were queried for the 5 driver variants of the gene based signals of SVEP1, BCO1, NELFA and IDH3A identified by SKAT **(Supplementary Table 10)**. The PheWAS query was limited to blood and cardiovascular traits as described in the methods. There were a total of 71 PheCodes, and each was looked up in African American (AA) and European American (EA) PheWAS from the BioVU Biobank, and UKBB as a whole. All PheCodes with a p<0.05 are listed in the table, and within each locus PheCodes are sorted by p-value. P-values are from a two-sided score test with no adjustment for multiple testing.

LOCUS (SNV_Effect allele)	PheCode	Description	BioVU						UKBB				
			OR	p-value	N cases	N controls	allele frequency	BioVU Population	OR	p-value	N cases	N controls	allele frequency
BC01 (rs143238312_C)	433.12	Cerebral atherosclerosis							4.055	0.003	224	399017	1.30%
	578.2	Blood in stool	0.557	0.047	899	17882	1.10%	EA					
	635	Hemorrhage during pregnancy; childbirth and postpartum	5.941	0.0012	37	22941	1.20%	EA					
	818	Intracranial hemorrhage (injury)	1.797	0.033	327	22278	1.20%	EA					
	818.1	Subdural hemorrhage (injury)	2.006	0.042	187	22282	1.20%	EA					
	430.2	Intracerebral hemorrhage							1.649	0.039	700	399017	1.30%
SVEP1 (rs61751937_C)	443.9	Peripheral vascular disease, unspecified							1.310	0.0015	2566	400595	2.90%
	443	Peripheral vascular disease							1.197	0.0088	3927	400595	2.90%
	578.2	Blood in stool							1.221	0.017	2639	385157	2.90%
	444	Arterial embolism and thrombosis	6.144	0.0055	48	4449	0.54%	AA	1.391	0.018	921	400595	2.90%
	444.1	Arterial embolism and thrombosis of lower extremity artery							1.522	0.018	557	400595	2.90%
	433	Cerebrovascular disease							1.116	0.02	NA	NA	2.90%
	433.11	Occlusion of cerebral arteries, with cerebral infarction	0.184	0.017	184	17494	2.70%	EA	1.116	0.02	NA	NA	2.90%
	433.2	Occlusion of cerebral arteries	2.887	0.036	166	4432	0.57%	AA					
	433.21	Cerebral artery occlusion, with cerebral infarction	3.218	0.022	150	4432	0.57%	AA					2.90%
	593	Hematuria							1.080	0.023	16409	379936	2.90%
	818	Intracranial hemorrhage (injury)	7.597	0.0084	33	5040	0.52%	AA					
	440	Atherosclerosis							1.284	0.032	1324	400595	2.90%
	411.8	Other chronic ischemic heart disease, unspecified							1.077	0.044	14921	377103	0.85%
IDH3A (rs61752770_A)	452.2	Deep vein thrombosis (DVT)	1.644	0.046	789	15863	1.75%	EA					
	593.1	Gross Hematuria	17.486	0.023	35	3482	0.09%	AA					
	850	Hemorrhage or hematoma complicating a procedure							0.771	0.017	5329	394929	

Supplementary Table 8: Annotation of genome-wide significant variants in RGS18 locus. Annotation evidence for megakaryocyte (MK) elements arbitrarily labeled to indicate four independent enhancer regions (e11 – e14) falling within a common super enhancer (se11). P-values are from a two-sided score test with no adjustment for multiple testing for trait and from a linear model with no adjustment for multiple testing for eQTLs.

		Lowest P value for Trait			Encode Regulatory		MK specific Regulatory		Platelet RGS18 eQTL		
**snpID hg38**	**MAF**	**ADP**	**COL**	**EPI**	**DNAse**	**TFBS Cluster**	**Enhancer**	**Super Enhancer**	**Beta**	**Var**	**P-value**
1:192139135:A:G	0.456	1.16E-05	4.42E-02	2.51E-08	Yes	GATA1	.	.	0.06	0	3.13E-02
1:192140500:T:A	0.43	3.54E-06	2.54E-02	2.17E-08	Yes	.	.	.	0.06	0	3.13E-02
1:192141301:A:C	0.43	2.55E-06	2.51E-02	1.81E-08	No	.	.	.	0.06	0	3.13E-02
1:192142107:T:TG	0.466	1.56E-05	5.81E-02	1.45E-08	No	.	.	.	.	.	.
1:192144114:G:A	0.437	3.34E-06	8.53E-02	4.57E-08	No	.	.	.	0.06	0	2.71E-02
1:192148472:C:T	0.465	1.17E-05	4.08E-02	1.55E-08	Yes	FOS|CEBPB	e11	se11	0.06	0	3.13E-02
1:192154581:A:G	0.449	2.79E-06	3.57E-02	1.79E-08	Yes	.	e12	se11	0.06	0	3.18E-02
1:192168015:T:A	0.445	9.49E-06	4.20E-02	6.98E-09	Yes	CTCF	e13	se11	0.06	0	2.73E-02
1:192174246:T:G	0.41	5.36E-07	5.55E-02	2.76E-08	No	.	e14	se11	0.07	0	1.40E-02
1:192182044:A:C	0.443	1.81E-05	3.71E-02	4.88E-09	No	.	.	.	0.08	0	2.29E-03
1:192183215:C:A	0.406	1.14E-06	5.63E-02	3.67E-08	No	.	.	.	0.08	0	1.83E-03
1:192186870:T:C	0.405	2.02E-06	5.64E-02	2.37E-08	Yes	.	.	.	0.08	0	1.83E-03
1:192186916:G:A	0.405	9.19E-07	6.08E-02	4.61E-08	Yes	.	.	.	0.09	0	1.01E-03
1:192193726:T:C	0.407	1.11E-06	5.35E-02	1.84E-08	No	.	.	.	0.08	0	1.83E-03
1:192193819:T:C	0.424	3.79E-06	3.76E-02	6.05E-09	No	.	.	.	0.08	0	2.29E-03
1:192194880:G:C	0.442	7.86E-06	2.37E-02	1.96E-09	Yes	.	.	.	0.08	0	2.29E-03
1:192195284:T:C	0.443	1.23E-05	3.47E-02	2.34E-09	No	.	.	.	0.08	0	2.38E-03
1:192206244:C:T	0.441	5.94E-07	1.32E-02	6.63E-09	Yes	E2F1	.	.	0.06	0	9.15E-03

Supplementary Table 9: Epigenetic annotation results underlying platelet SNP selection for functional enhancer activity screens with transcription factor overexpression as well as summary results of assay screens.

**Locus**	**SNPid**	**Luciferase screen result**	**SNPchr:pos:alleles**	**minP (platelet agg traits)**	**K562 TFBS**	**ATAC-Seq (MKs)***	**ENC_Dnase**	**hot_MK**	**ENCODE TFBS (all cell types)**	**Superenhancer _MK (Blueprint)**	**h3k27ac_MK (Blueprint)****	**h3k4me3_MK (Blueprint)****	**h3k4me3.prom (promoter bound in MKs)**
PEAR1	rs12041331	Insufficent enhancer activity	1:156899922:G:A	2.31E-18	NRF1, NRF1	.	239	.	.	chr1.156865800.mkse	.	.	.
PEAR1	rs12566888	Insufficent enhancer activity	1:156899255:G:T	5.73E-14	BCOR, KDM1A, KDM1A, ZMYM3	.	371	.	POLR2A	chr1.156865800.mkse	2	.	.
PEAR1	rs12086222	Insufficent enhancer activity	1:156899838:G:C	2.17E-07	.	.	239	.	.	chr1.156865800.mkse	1	.	.
PEAR1	rs822442	No allele-specific or TF (CTCF) effect in HEK293 or K562 cells	1:156913423:C:A	3.07E-06	ARID1B, ARNT, ATF3, ATF7, CBFA2T3, CTBP1, CTCF, DPF2, GATAD2A, HDAC1, HDAC1, HNRNPL, KDM1A, LEF1, MAX, MGA, MLLT1, MNT, MTA2, MYNN, NBN, NCOR1, NFXL1, NR2F1, NRF1, NRF1, POLR2A, RB1, RBFOX2, RNF2, RNF2, SKIL, SMARCA4, ZEB2, eGFP-ATF1, eGFP-DIDO1, eGFP-E2F5, eGFP-ETV1, eGFP-FOSL1, eGFP-VEZF1, eGFP-ZBTB11, eGFP-ZNF395, DPF2, HDAC2, L3MBTL2, PKNOX1, POLR2G, ZEB2, eGFP-ZFX, eGFP-ZNF148	5	692	.	ZNF263, MAZ, ZBTB7A, SAP30, CTCF, E2F6, RAD21, POLR2A, HMGN3, PHF8	chr1.156865800.mkse	.	1	.
RGS18	rs1175170	Insufficent enhancer activity	1:192194880:G:C	1.96E-09	.	.	.	.	.	.	.	.	.
RGS18	rs10754003	No allele-specific or TF (CTCF) effect in HEK293 or K562 cells	1:192168015:T:A	6.98E-09	.	.	693	2	CTCF	chr1.192116200.mkse	4	.	.
RGS18	**rs6687273**	**No allele-specific or TF (CEBPB) effect in HEK293. Reduction of enhancer activity with T allele in K562 cells**	**1:192148472:C:T**	**1.55E-08**	**eGFP-NFE2**	**8**	**1000**	**2**	**FOS, CEBPB**	**chr1.192116200.mkse**	**2**	**.**	**.**
RGS18	**rs12070423**	**G allele reduces enhancer activity in GATA1 overexpressing HEK293 and K562 cells**	**1:192139135:A:G**	**2.51E-08**	**.**	**.**	**.**	**.**	**GATA1**	**.**	**.**	**.**	**.**
RGS18	**rs4495675**	**G allele reduces enhancer activity in NFE2 overexpressing HEK293 and K562 cells**	**1:192158507:T:G**	**8.30E-08**	**NFE2, TEAD4, eGFP-CEBPB, eGFP-CEBPG**	**8**	**.**	**2**	**POLR2A, SPI1, BATF**	**chr1.192116200.mkse**	**4**	**2**	**NM_130782**
ADRA2A	rs7097060	No allele-specific or TF (FOSL1) effect in HEK293 or K562 cells	10:111139289:T:A	6.68E-12	eGFP-FOSL1	.	.	.	.	.	.	.	.
ADRA2A	rs10885088	Insufficent enhancer activity	10:111114449:A:G	2.06E-11	.	8	1000	4	POLR2A	chr10.112872600.mkse	2	1	.
ADRA2A	rs7079429	Insufficent enhancer activity	10:111123099:T:C	2.75E-10	.	.	.	.	.	chr10.112872600.mkse	.	.	.
ADRA2A	rs12161672	Insufficent enhancer activity	10:111127713:C:G	4.95E-10	ARID1B, CBFA2T2, CBFA2T3, NCOR1, NCOR1, NR2F2, STAT5A, TAL1, TAL1, TCF12, TEAD4, TRIM24, eGFP-VEZF1, eGFP-ZNF589	8	621	6	GATA2, POLR2A, STAT5A, TEAD4, NR2F2, GATA1, TAL1	chr10.112872600.mkse	.	1	.

Column heading descriptions.

Locus = major gene in region under study
SNP = Platelet aggregaton associateed SNP
Luciferase assay results = summary of attempted enhancer activity assays
Chr:pos:allele1:allele2 = location/major/minor alleles
minP (platelet aggregation traits) = lowest observed p-value for platelet aggregation traits in the GWAS
K562.TFBS = annotation of overlap of variants with 304 TF datasets from ENCODE K562 cells
ATAC-seq MKS = overlap with megakaryocyte ATAC-seq peaks in Blueprint
ENC_DNAse = stringent definition of DNAse cluster aross all cell types
hot_MK = DNAse hotspot peaks in megakaryocytes
ENCODE TFBS = overlapping TF clusters across all ENCODE cell types
Superenhancer_MK = overlapping superenhancer in megakaryocytes
H3K27ac MK = overlapping H3K27ac peak in megakaryocytes
H3K4me3_MK = overlapping H3K4me3 peak in megakaryocytes
H3K4me3 bound = transcript with bound H3K4me3 overlapping promoter peak in megakaryocytes

* total number of clones displaying open chromatin in the region in MKs
** total number of samples with overlapping feature

Supplementary Table 10: Aggregated rare deleterious coding variants of 4 genes (SVEP1, BCO1, NELFA and IDH3A) were associated with platelet aggregation after Bonferroni correction (0.05 / 17744 = 2.819E-6) by SKAT with MAF threshold 0.05. P-values are from a two-sided score test with no adjustment for multiple testing.

Gene	Chr	Start	Stop	# variants	p-value	Phenotype (see Supplemetnary Table 1
SVEP1	9	110365251	110579880	64	2.64E-06	adp_low1
BCO1	16	81238448	81291142	27	8.88E-07	epi_low1
NELFA	4	1982714	2041903	11	1.70E-06	col_high1
IDH3A	15	78131498	78171949	10	2.40E-06	col_high2

Supplementary Table 11: Annotation of 5 variants driving signals of SVEP1, BCO1, NELFA and IDH3A identified by SKAT gene-based test with MAF threshold 0.05.

Chr:pos	Ref/Alt	Gene	AA Change	dbSNP	SIFT	P2 HVAR	LRT	Mutation Taster	Meta SVM	M-CAP	CADD	REVEL
16:81290348	G/C	BCO1	p.G472A	rs143238312	D	D	D	D	D	.	25.2	0.875
9:110549951	G/C	SVEP1	p.R229G	rs61751937	D	D	D	D	D	.	28.3	0.672
4:1986122	T/C	NELFA	p.K287R	rs150291014	T	B	N	D	T	D	15.89	0.062
4:1987948	G/A	NELFA	p.R213W	rs763817905	D	D	D	D	D	D	35	0.457
15:78161708	T/A	IDH3A	p.D139E	rs61752770	D	P	D	D	T	D	24.7	0.208

Supplementary Table 12. Oligonucleotides used in functional experiments.

qRT-PCR	Assay ID	Probe	Company
POLR2A	Hs.PT.58.25515089	FAM	IDT-DNA
NRF1	Hs.PT.58.19519028	FAM	IDT-DNA
CTCF	Hs.PT.58.27300879	FAM	IDT-DNA
FOSL1	Hs.PT.58.2855727	FAM	IDT-DNA
GATA1	Hs.PT.58.21050378	FAM	IDT-DNA
GATA2	Hs.PT.58.961996	FAM	IDT-DNA
CEBPB	Hs.PT.58.27185099	FAM	IDT-DNA
NFE2	Hs.PT.58.50438577	FAM	IDT-DNA
Beta-Actin	Hs.PT.39a.22214847	HEX	IDT-DNA
PGL3 vectors			
Gene	Wildtype	SNPs	SNP-ID
RGS18	rs1175170 (G)	rs1175170 (C)	1:192194880:G:C
RGS18	rs10754003 (T)	rs10754003 (A)	1:192168015:T:A
RGS18	rs6687273 (C)	rs6687273 (T)	1:192148472:C:T
RGS18	rs12070423 (A)	rs12070423 (G)	1:192139135:A:G
RGS18	rs4495675 (T)	rs4495675 (G)	1:192158507:T:G
ADRA2A	rs7097060 (T)	rs7097060 (A)	10:111139289:T:A
ADRA2A	rs10885088 (A)	rs10885088 (G)	10:111114449:A:G
ADRA2A	rs7079429 (T)	rs7079429 (C)	10:111123099:T:C
ADRA2A	rs12161672 (C)	rs12161672 (G)	10:111127713:C:G
PEAR1	rs12041331 (G)	rs12041331 (A)	1:156899922:G:A
PEAR1	rs12086222 (G)	rs12086222 (C)	1:156899838:G:C
PEAR1	rs12566888 (G)	rs12566888 (T)	1:156899255:G:T
PEAR1	rs822442 (C)	rs822442 (A)	1:156913423:C:A

Supplementary Figure 1. Manhattan plots and Quantile-Quantile (QQ) plots of platelet aggregation phenotypes.

Supplementary Figure 1. Manhattan plots and Quantile-Quantile (QQ) plots of platelet aggregation in response to different doses of Epinephrine, ADP and Collagen as described in Supplementary Table 1. Genome-wide association study for platelet aggregation in 3,855 individuals. P-values, expressed as -log 10(P), are plotted according to physical genomic locations by chromosome. Loci passing genome wide significance (5×10^{-8}) are marked by red dots. Locus names represent the nearest annotated gene. P-values are from a two-sided score test with no adjustment for multiple testing. The blue horizontal line indicates a p-value threshold of 1×10^{-6} corresponding to suggestive significance threshold. The red horizontal line indicates p-value threshold of 5×10^{-8}, corresponding to genome-wide significance.

ADP low1

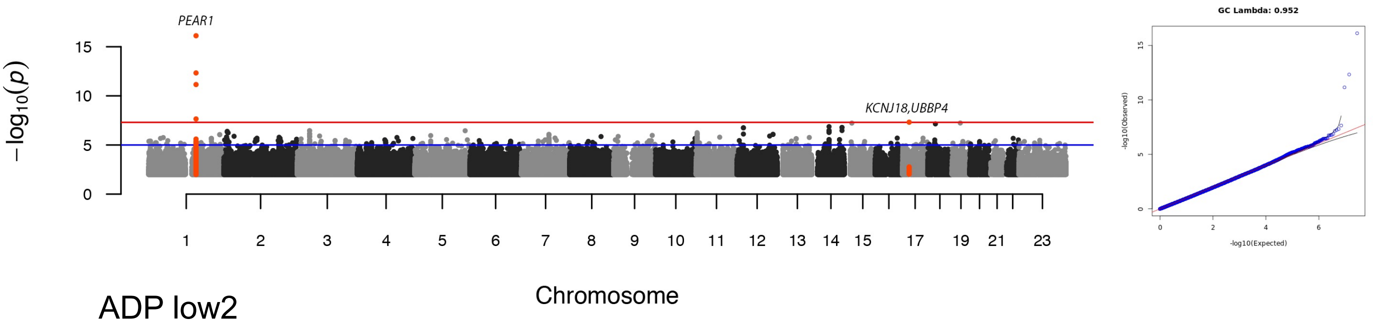

ADP low2

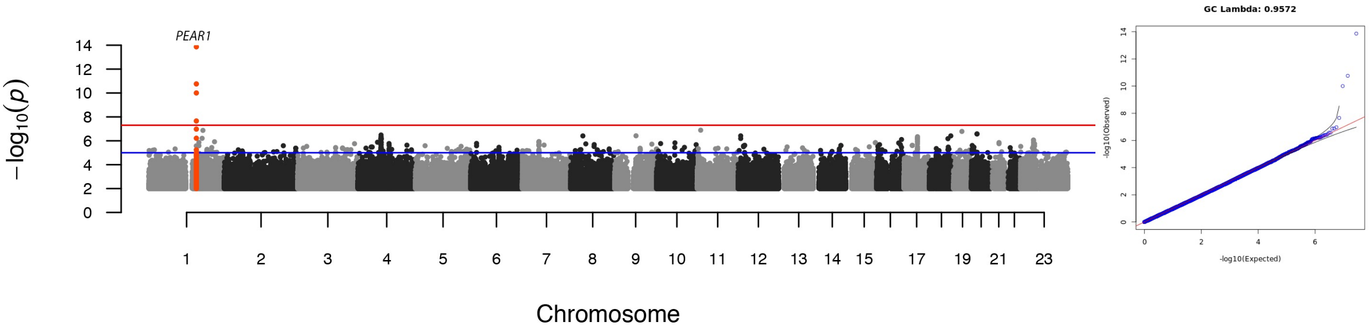

ADP low3

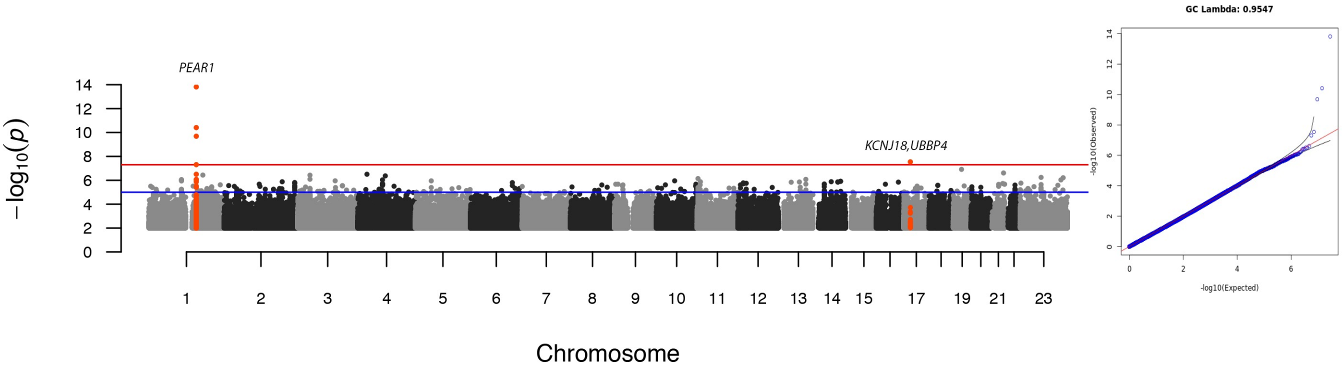

Supplementary Figure 1 continued.

ADP high1

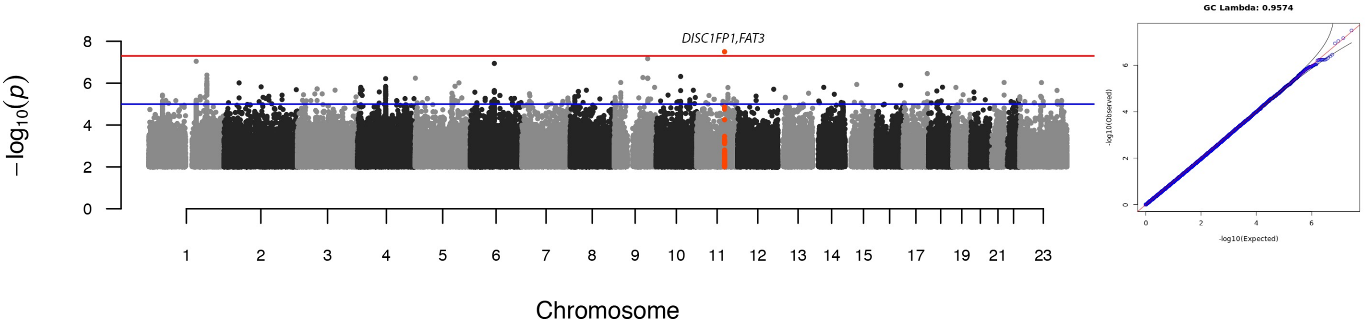

ADP high2

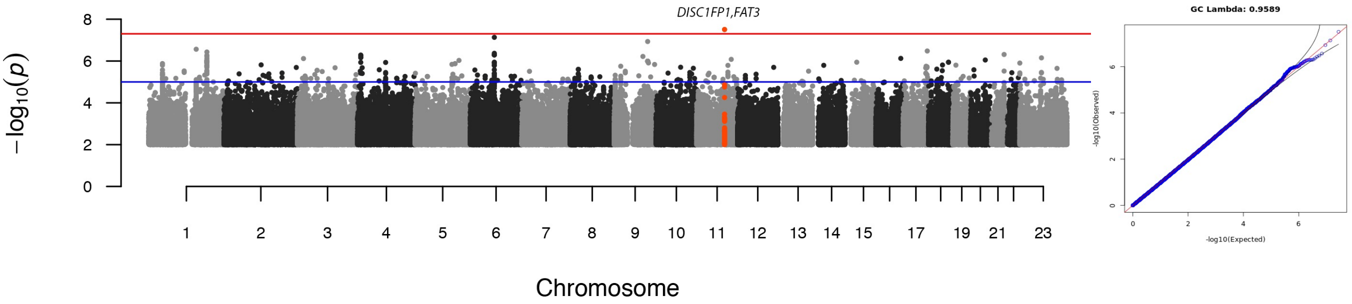

ADP high3

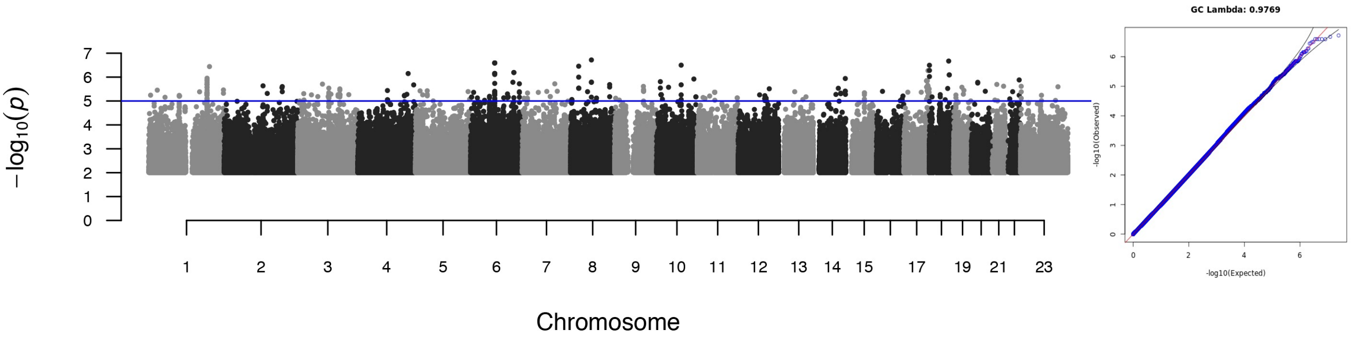

ADP high4

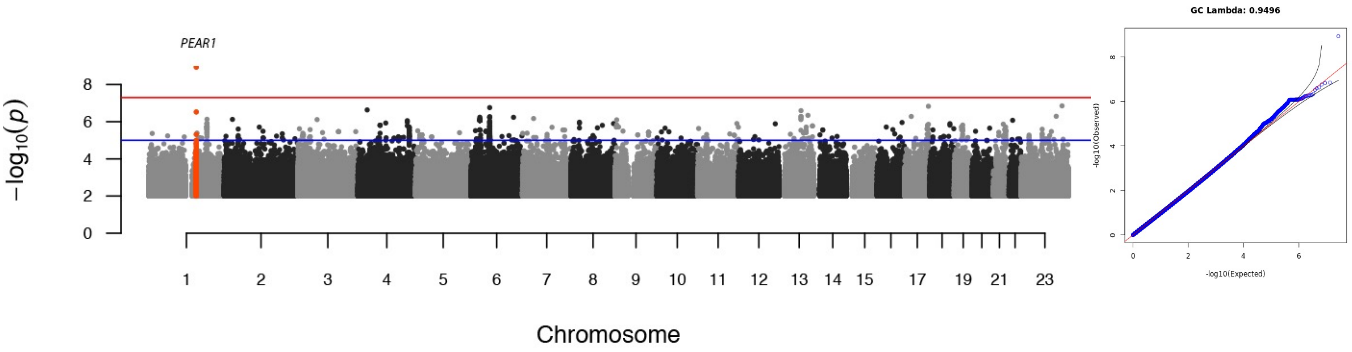

Supplementary Figure 1 continued.

Collagen low1

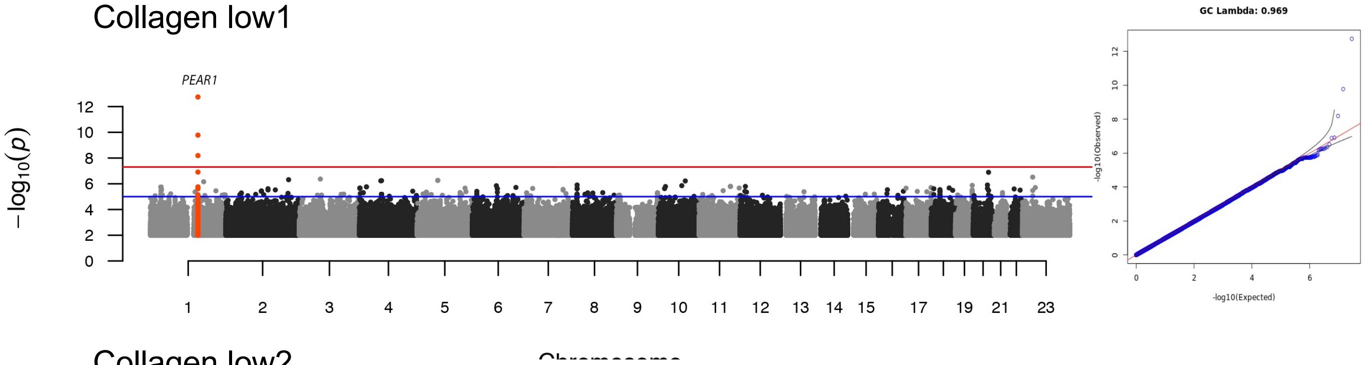

Collagen low2

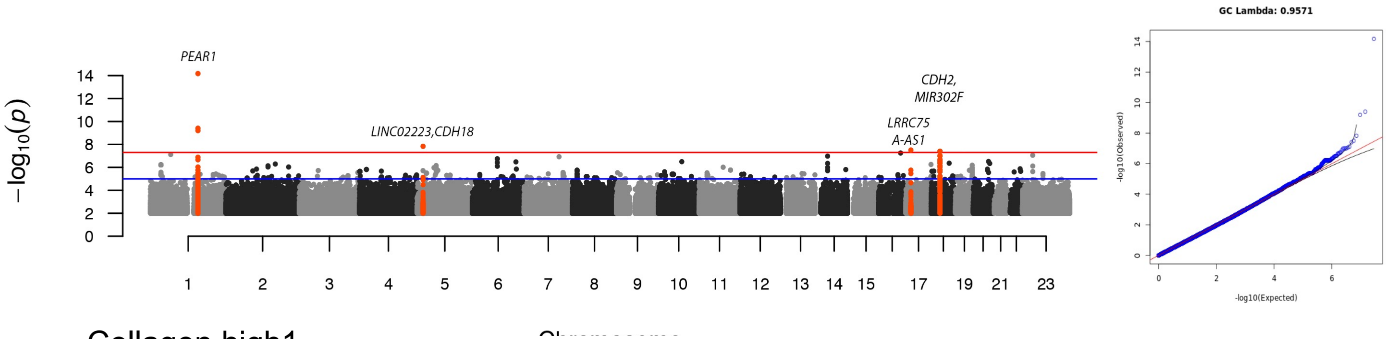

Collagen high1

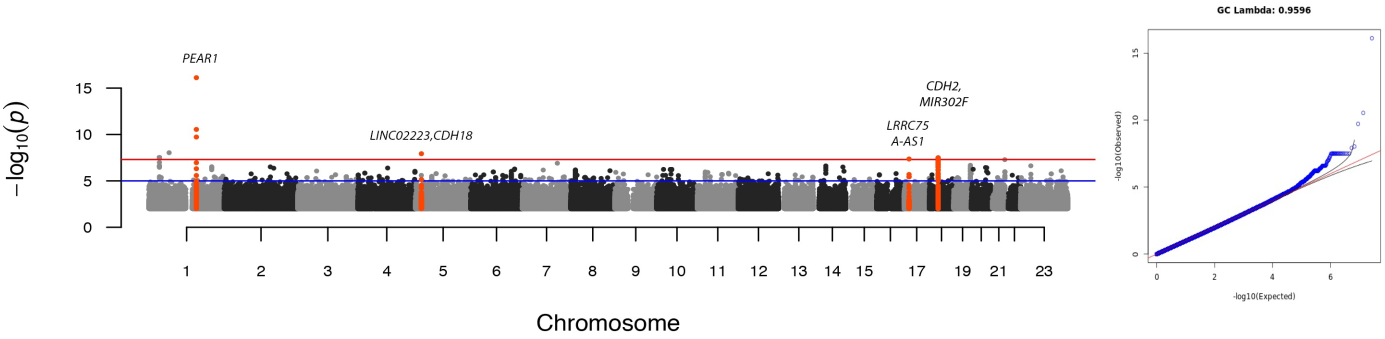

Collagen high2

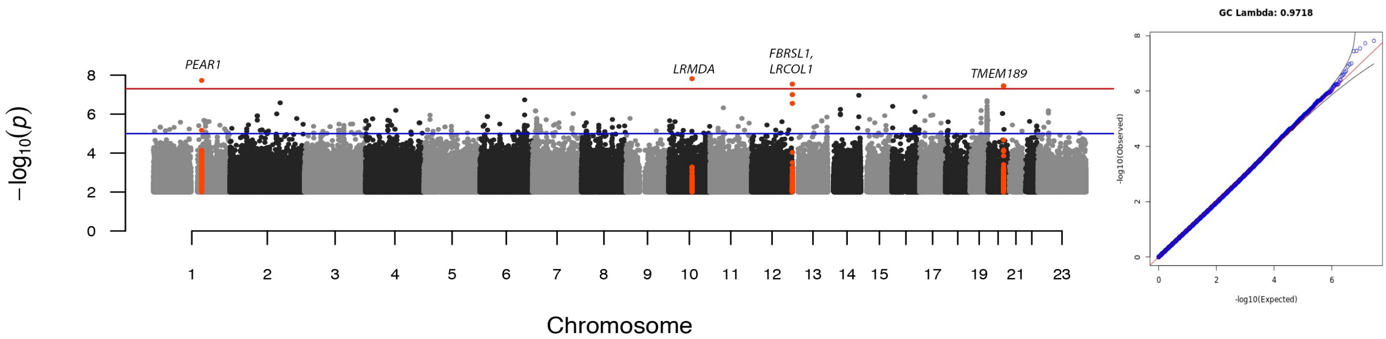

Supplementary Figure 1 continued.

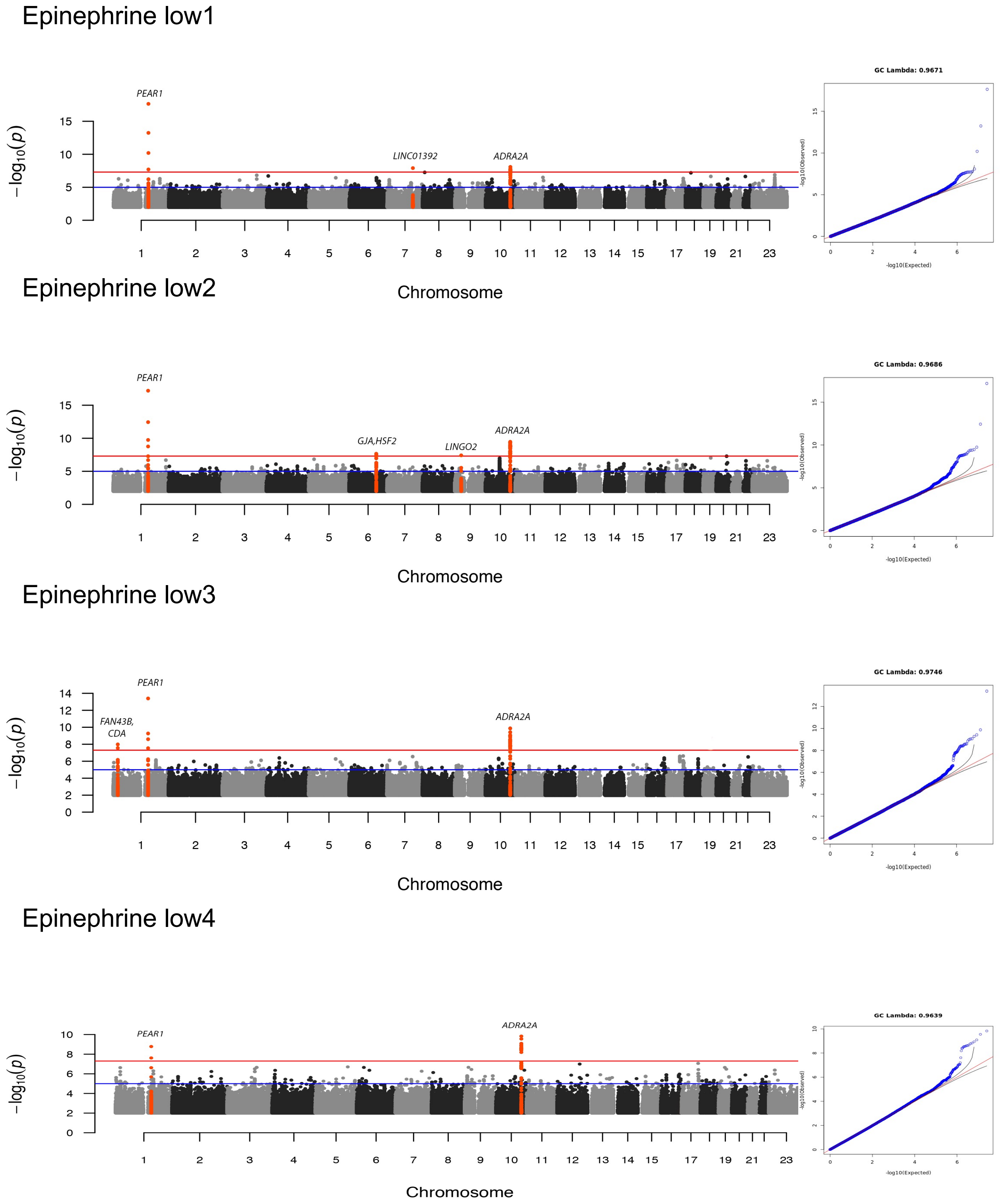

Supplementary Figure 1 continued.

Epinephrine low5

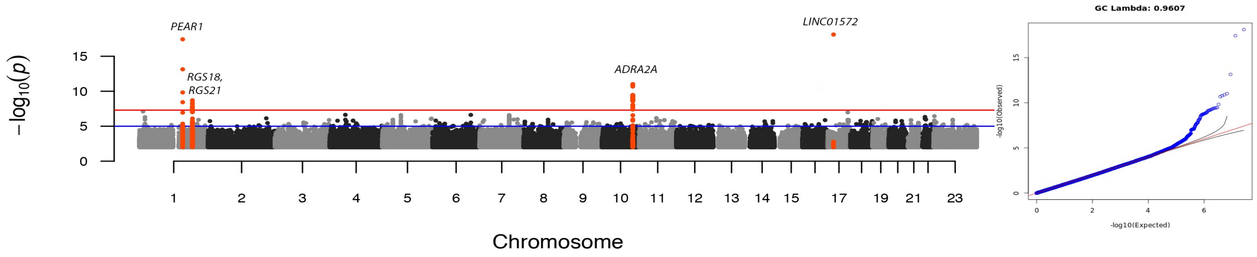

Epinephrine high1

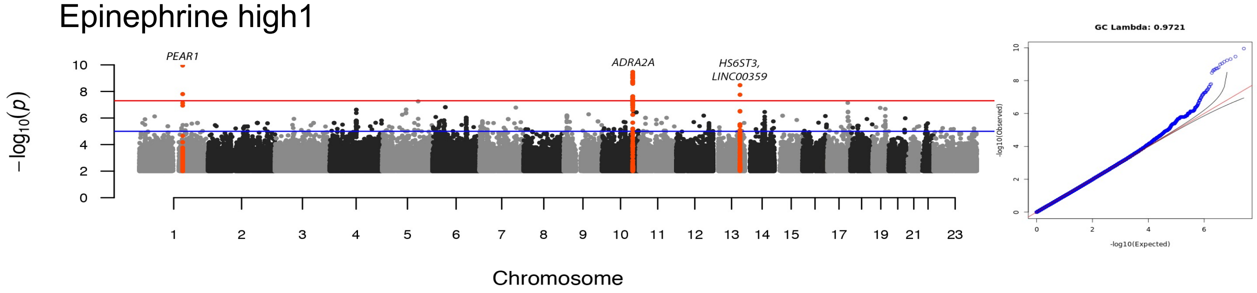

Epinephrine high2

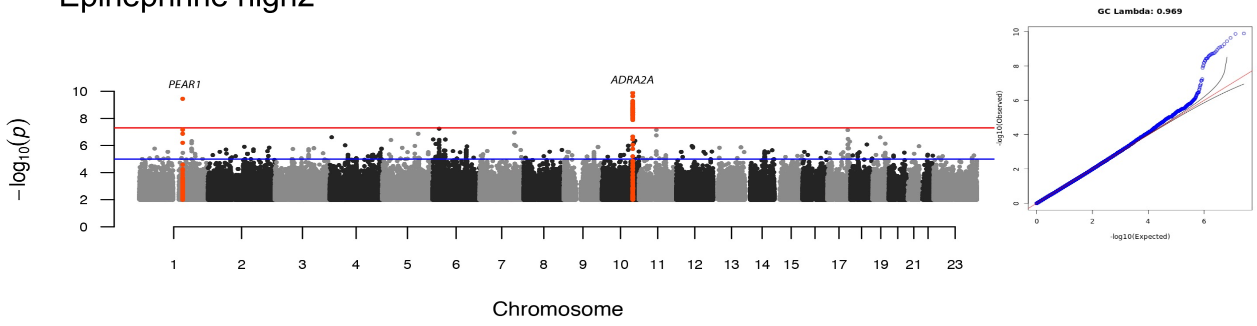

Epinephrine high3

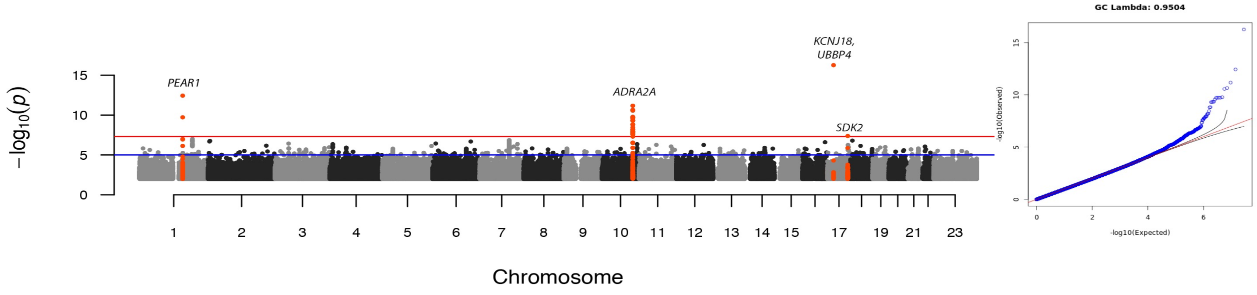

Supplementary Figure 2A: Box plots of ADP-Induced Platelet Reactivity phenotypes for sentinel GWAS variants.

Supplementary Figure 2A: Association between ADP-Induced Platelet Reactivity and Genome-Wide Significant Loci using Single-Variant Approaches. For each box plot, the horizontal line within each box indicates the median; the top and bottom borders of each box indicate the inter-quartile range. The whiskers extending from each box indicate the 95% confidence interval and individual data points are shown in red. Platelet reactivity is expressed as the inverse normalized transformations of residuals from linear models as described in the Methods section. For each polymorphism, the lowest p-value among ADP-based phenotypes is shown. P-values are from a two-sided score test with no adjustment for multiple testing from the GWAS analysis.

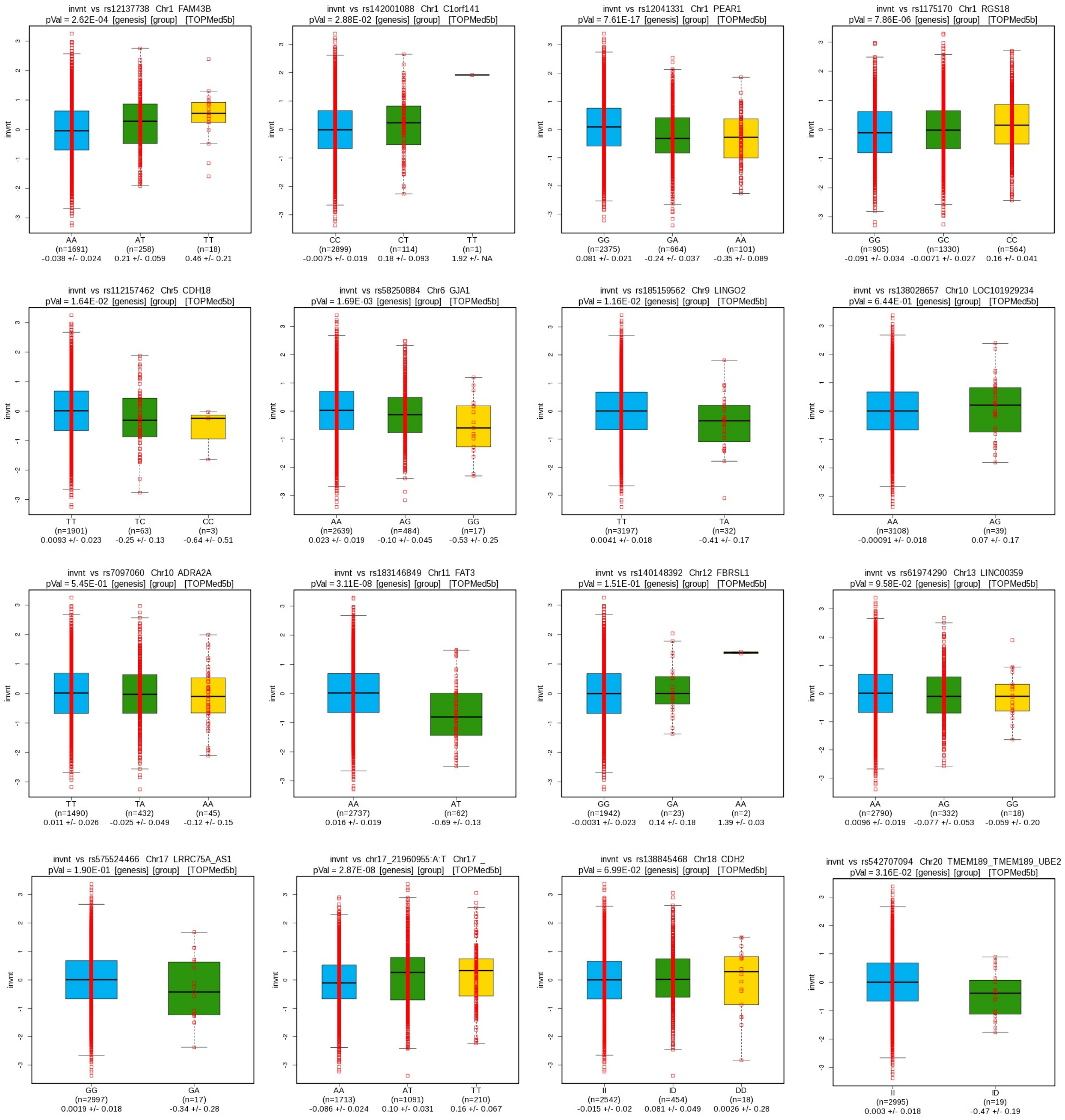

Supplementary Figure 2B: Box plots of Collagen-Induced Platelet Reactivity phenotypes for sentinel GWAS variants.

Supplementary Figure 2B: Association between Collagen-Induced Platelet Reactivity and Genome-Wide Significant Loci using Single-Variant Approaches. For each box plot, the horizontal line within each box indicates the median; the top and bottom borders of each box indicate the inter-quartile range. The whiskers extending from each box indicate the 95% confidence interval and individual data points are shown in red. Platelet reactivity is expressed as the inverse normalized transformations of residuals from linear models as described in the Methods section. For each polymorphism, the lowest p-value among collagen-based phenotypes is shown. P-values are from a two-sided score test with no adjustment for multiple testing from the GWAS analysis

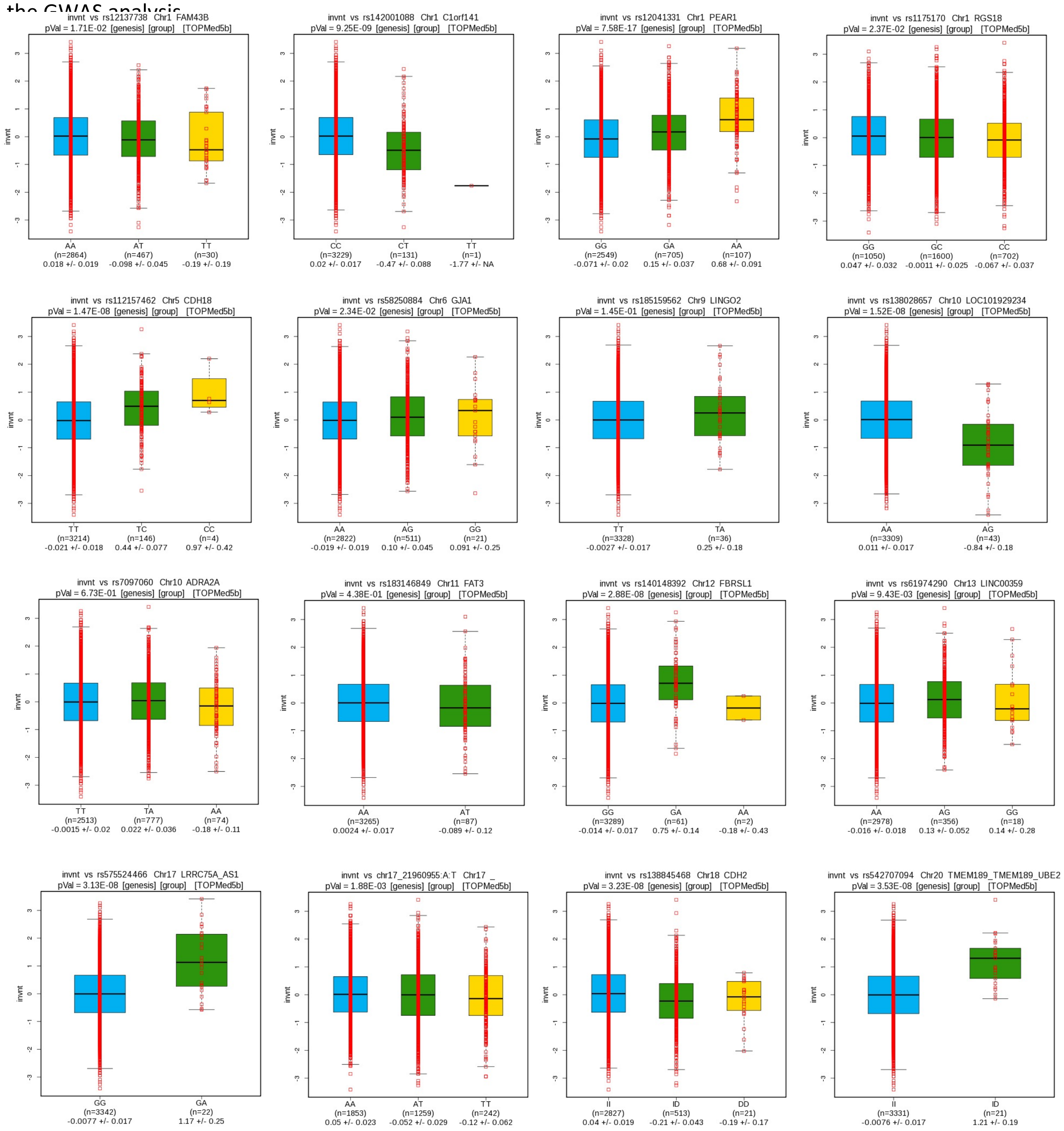

Supplementary Figure 2C: Box plots of Epinephrine-Induced Platelet Reactivity phenotypes for sentinel GWAS variants.

Supplementary Figure 2C: Association between Epinephrine-Induced Platelet Reactivity and Genome-Wide Significant Loci using Single-Variant Approaches. For each box plot, the horizontal line within each box indicates the median; the top and bottom borders of each box indicate the inter-quartile range. The whiskers extending from each box indicate the 95% confidence interval and individual data points are shown in red. Platelet reactivity is expressed as the inverse normalized transformations of residuals from linear models as described in the Methods section. For each polymorphism, the lowest p-value among epinephrine-based phenotypes is shown. P-values are from a two-sided score test with no adjustment for multiple testing from the GWAS analysis.

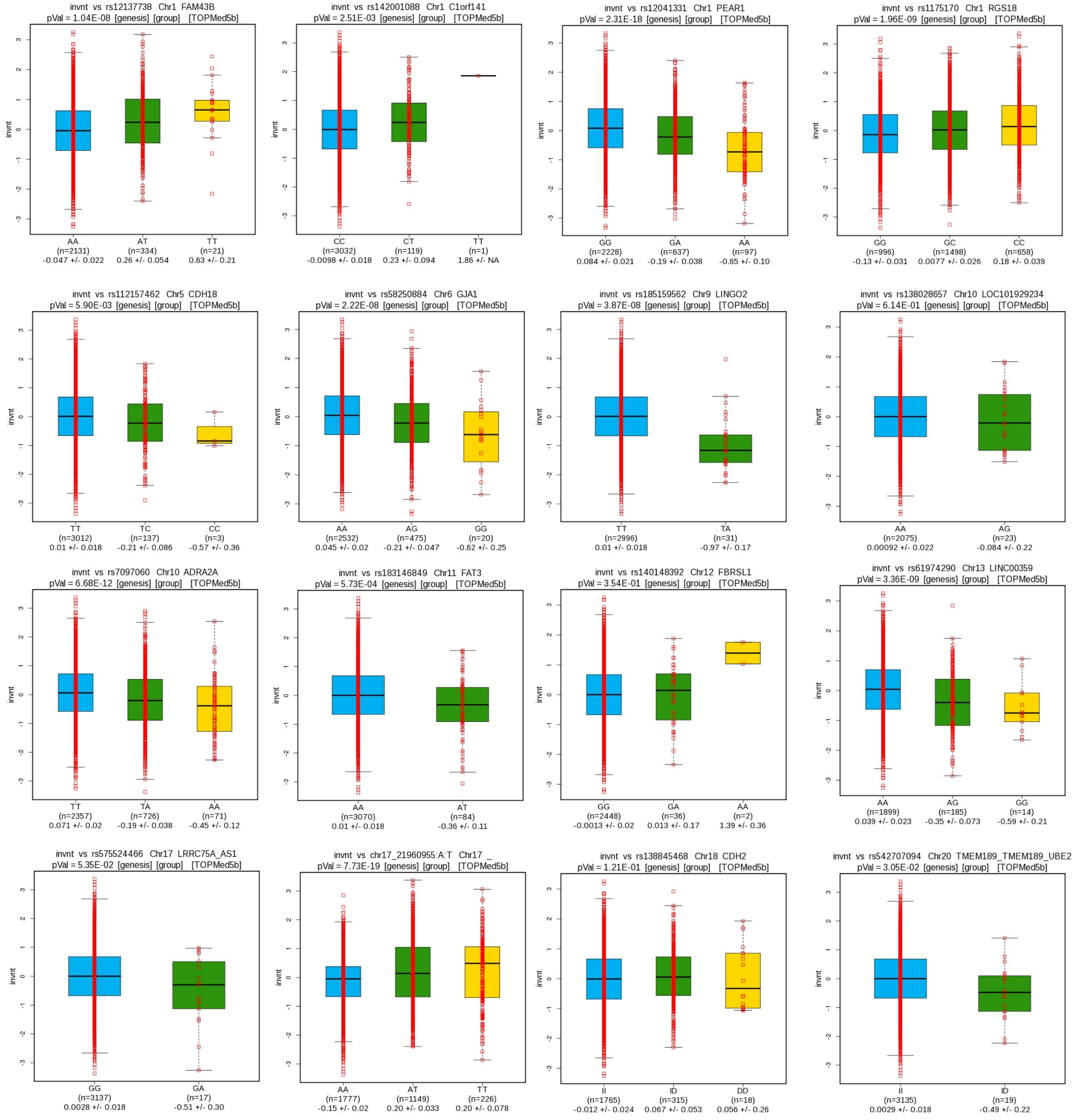

Supplementary Figure 3. Locus zoom plots of GWAS loci associated.

Supplementary Figure 3. Locus zoom plots of independent loci associated with platelet aggregation in response to [**A**] ADP, [**B**] Collagen(and [**C**] Epinephrine. P-values are from a two-sided score test with no adjustment for multiple testing from the GWAS analysis.

[A]

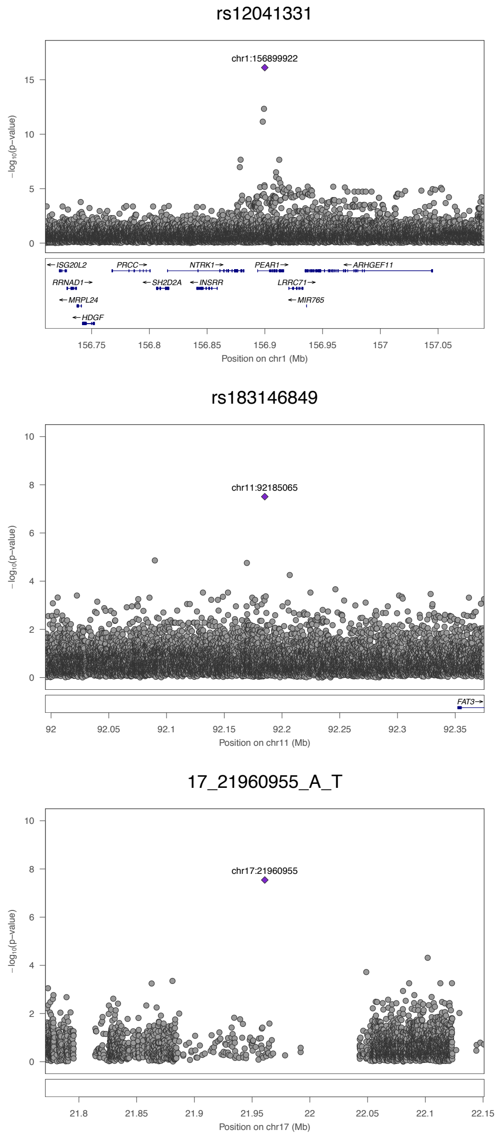

[B]

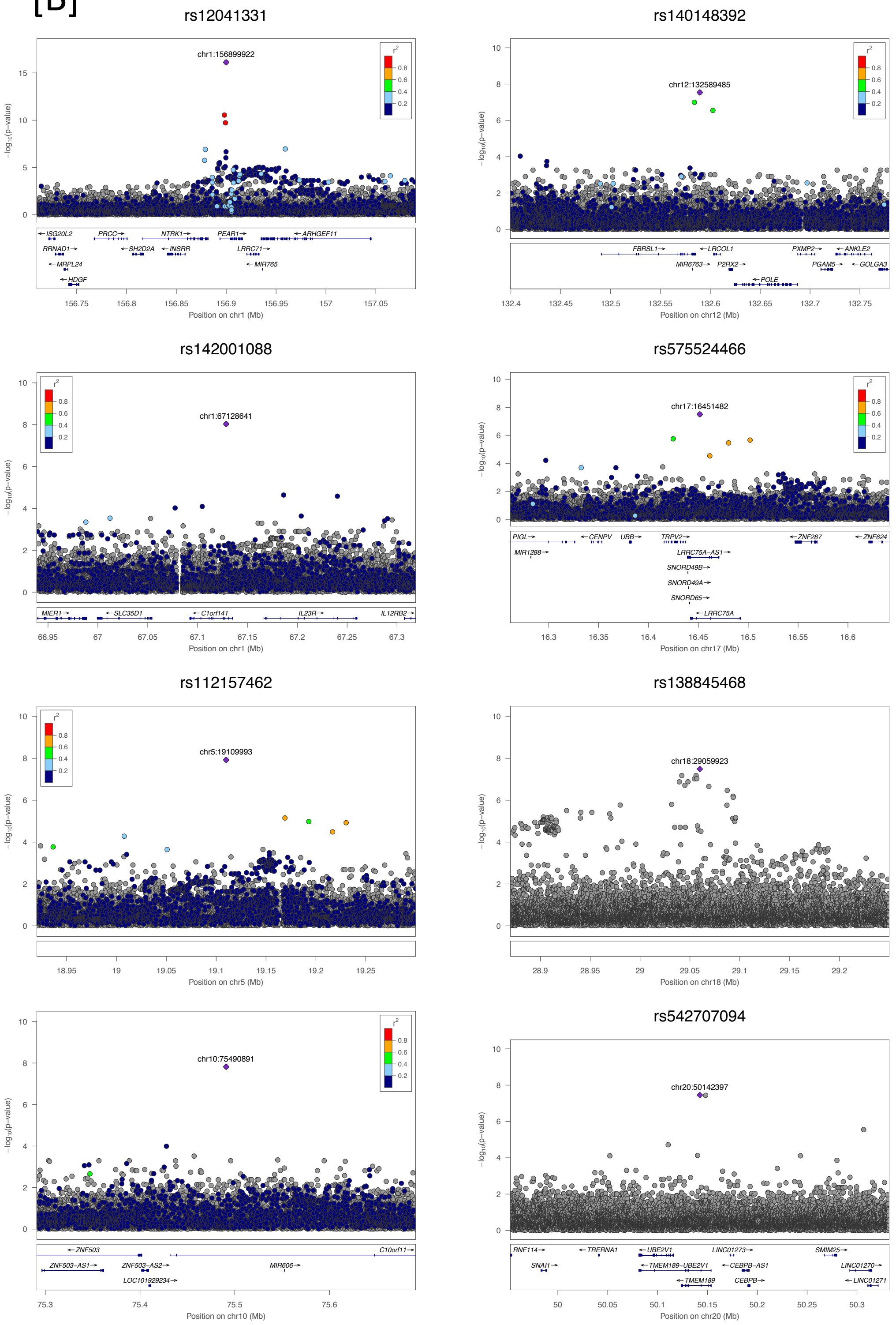

rs12041331
chr1:156899922
ISG20L2
PRCC
NTRK1
PEAR1
ARHGEF11
RRNAD1
SH2D2A
INSRR
LRRC71
MRPL24
MIR765
HDGF
Position on chr1 (Mb)
rs140148392
chr12:132589485
FBRSL1
LRCOL1
PXMP2
ANKLE2
MIR6763
P2RX2
PGAM5
GOLGA3
POLE
Position on chr12 (Mb)
rs142001088
chr1:67128641
MIER1
SLC35D1
C1orf141
IL23R
IL12RB2
Position on chr1 (Mb)
rs575524466
chr17:16451482
PIGL
CENPV
UBB
TRPV2
ZNF287
ZNF624
MIR1288
LRRC75A−AS1
SNORD49B
SNORD49A
SNORD65
LRRC75A
Position on chr17 (Mb)
rs112157462
chr5:19109993
Position on chr5 (Mb)
rs138845468
chr18:29059923
Position on chr18 (Mb)
chr10:75490891
ZNF503
C10orf11
ZNF503−AS1
ZNF503−AS2
MIR606
LOC101929234
Position on chr10 (Mb)
rs542707094
chr20:50142397
RNF114
TRERNA1
UBE2V1
LINC01273
SMIM25
SNAI1
TMEM189−UBE2V1
CEBPB−AS1
LINC01270
TMEM189
CEBPB
LINC01271
Position on chr20 (Mb)
−log10(p−value)
r2

[C]

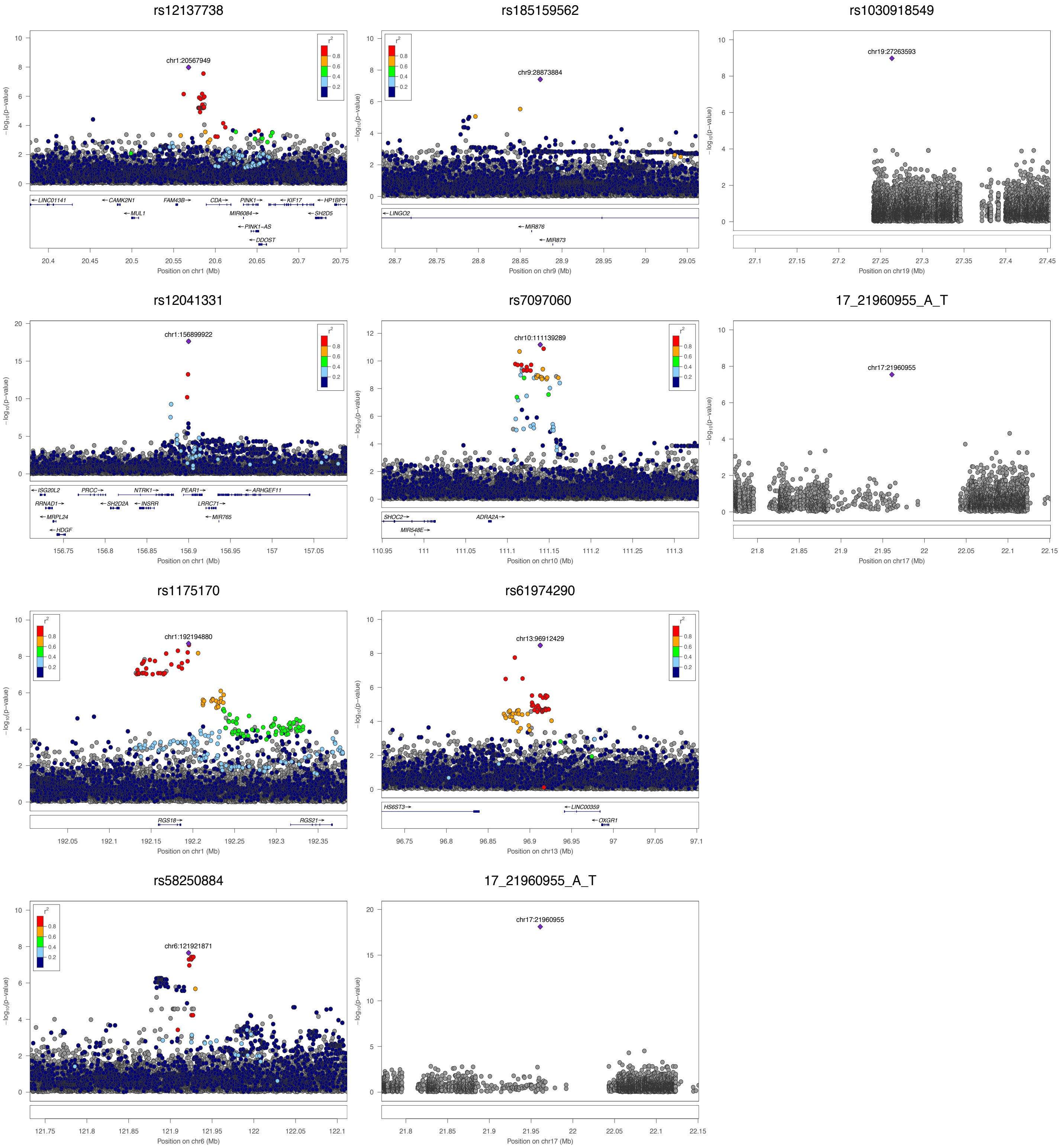
rs12137738
chr1:20567949
−LINC01141
←CAMK2N1
FAM43B→
CDA→
PINK1→
←KIF17
←HP1BP3
←MUL1
MIR6084→
←SH2D5
←PINK1−AS
←DDOST
Position on chr1 (Mb)
−log10(p−value)
rs185159562
chr9:28873884
←LINGO2
←MIR876
←MIR873
Position on chr9 (Mb)
rs1030918549
chr19:27263593
Position on chr19 (Mb)
rs12041331
chr1:156899922
←ISG20L2
PRCC→
NTRK1→
PEAR1→
←ARHGEF11
RRNAD1→
←SH2D2A
←INSRR
LRRC71→
←MRPL24
←MIR765
←HDGF
Position on chr1 (Mb)
rs7097060
chr10:111139289
SHOC2→
ADRA2A→
MIR548E→
Position on chr10 (Mb)
17_21960955_A_T
chr17:21960955
Position on chr17 (Mb)
rs1175170
chr1:192194880
RGS18→
RGS21→
Position on chr1 (Mb)
rs61974290
chr13:96912429
HS6ST3→
←LINC00359
←OXGR1
Position on chr13 (Mb)
rs58250884
chr6:121921871
Position on chr6 (Mb)
17_21960955_A_T
chr17:21960955
Position on chr17 (Mb)
r2
0.8
0.6
0.4
0.2

Supplementary Figure 4: Evaluation of allele-specific enhancer activity.

Supplementary Figure 4: Evaluation of allele-specific enhancer activity differences via mutagenesis of top prioritized SNPs that are associated with RGS18 (rs10754003, rs6687273, rs12070423, rs4495675) ADRA2A (rs7097060), PEAR1 (rs822442) in HEK293 cells overexpressing CTCF, CEPBP, GATA1, NFE2, and FOSL1 in **[A]** and in K562 Cells in **[B]**. P-values are from a two-sided Welch test with no adjustment for multiple testing. In panel A: 6 biological replicates over 3 independent experiments were used for the GFP control, 12 biological replicates over 3 independent experiments were used for the experimental transcription factors. In panel B, 24 biological replicates over 3 independent experiments were used. Bar plots depict the mean values +/- SEM

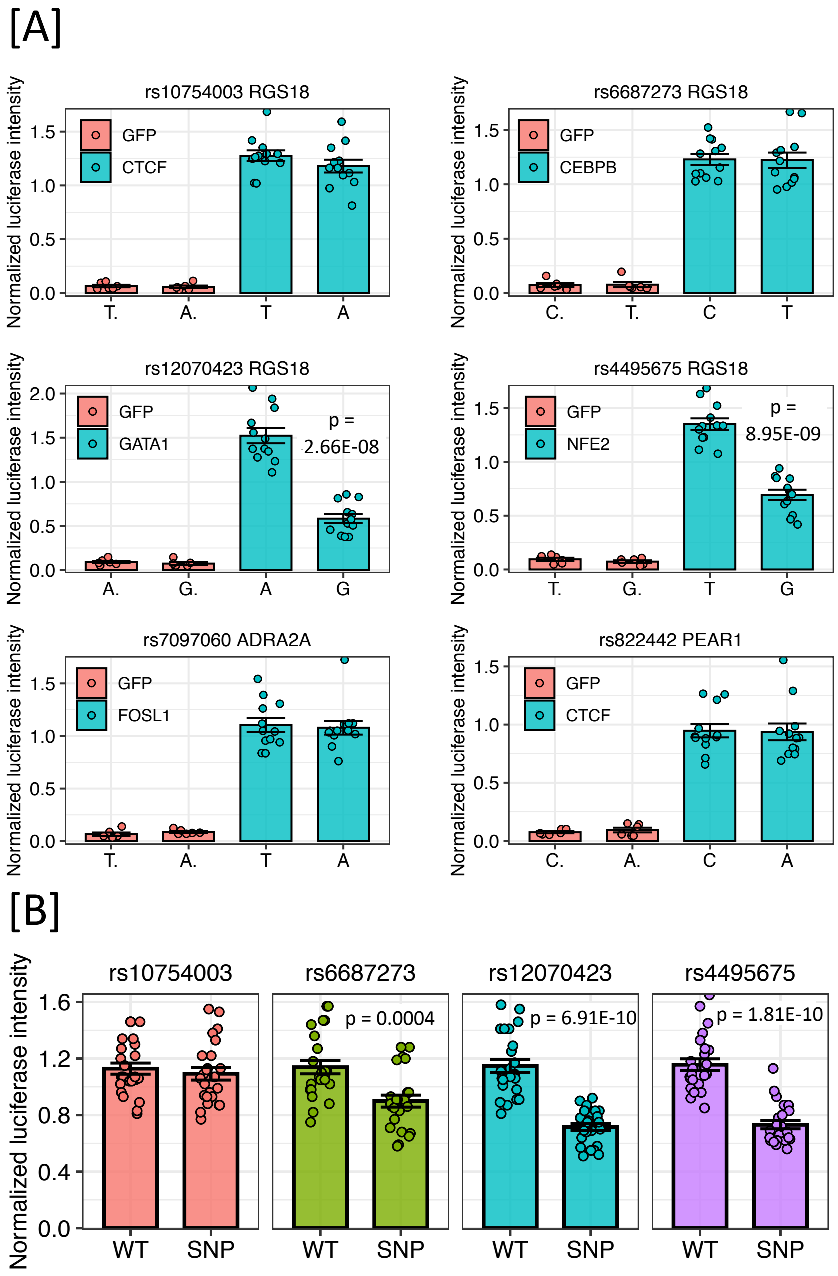

Supplementary Figure 5: Overexpression of various transcription factors in HEK-293 cells.

Supplementary Figure 5: Overexpression of various transcription factors in HEK-293 cells. HEK293 cells were transduced with lentiviral vector containing ORF of GFP (Control), POLR2A, NRF1, CTCF, FOSL1, GATA1, GATA2, CEPBP, and NFE2. Expression of various transcription factors mRNA were measured by RT-qPCR, normalized to β-actin.

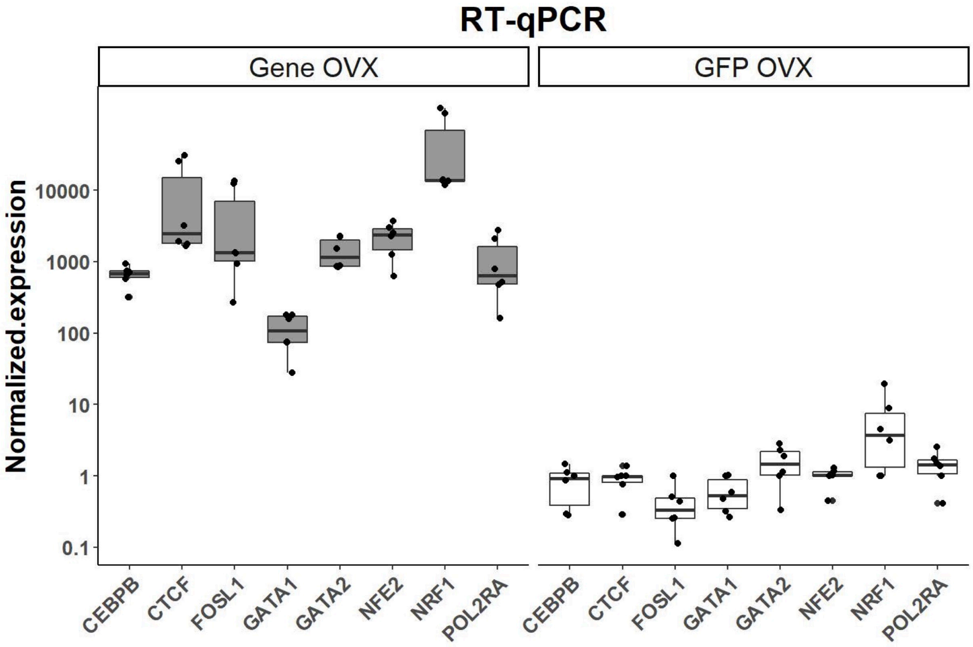

Supplementary Figure 6: QQ plots of SKAT analyses of rare deleterious coding variants.

Supplementary Figure 6: QQ plots of SKAT analyses of rare deleterious coding variants with ADP, Collagen and Epinephrine induced platelet aggregation. P-values are from a two-sided score test with no adjustment for multiple testing from the SKAT analysis.

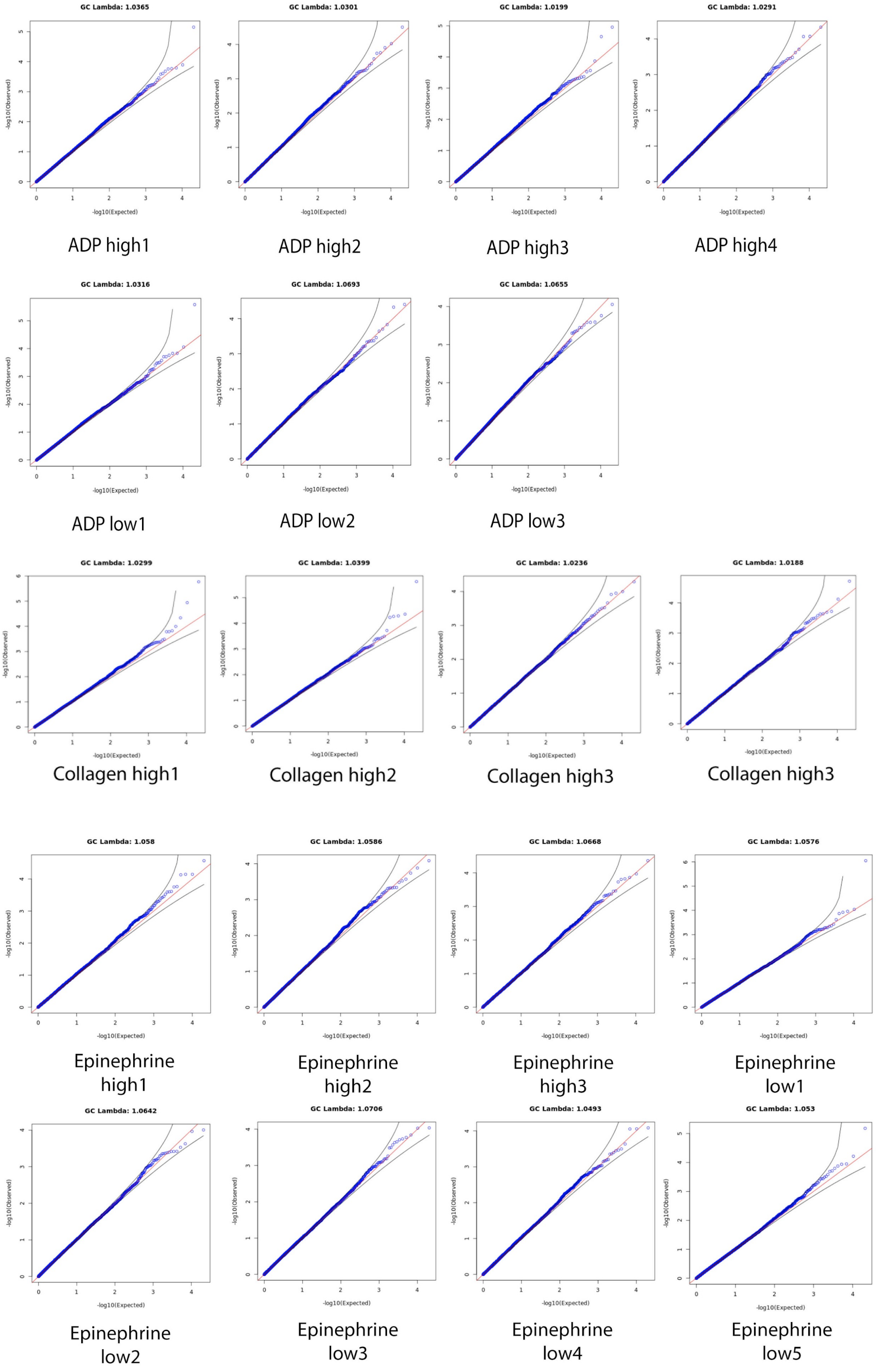

Supplementary Figure 7: Leave-one-out technique to identify the variants contributing the most to SKAT p-value.

Supplementary Figure 7: Leave-one-out technique to identify the variants contributing the most to SKAT p-value of [**A**] SVEP1, [**B**] BCO1, [**C**] NELFA and [**D**] IDH3A were associated with platelet aggregation after Bonferroni correction (0.05 / 17744 = 2.819x10^{-6}). X-axis represents the variants in the gene set, orange dots represent the –log10(p-value) of gene set when the variant was left out and blue bars represent the minor allele frequency of the variants. P-values are from two-sided score tests with no adjustment for multiple testing.

[A] SVEP1,

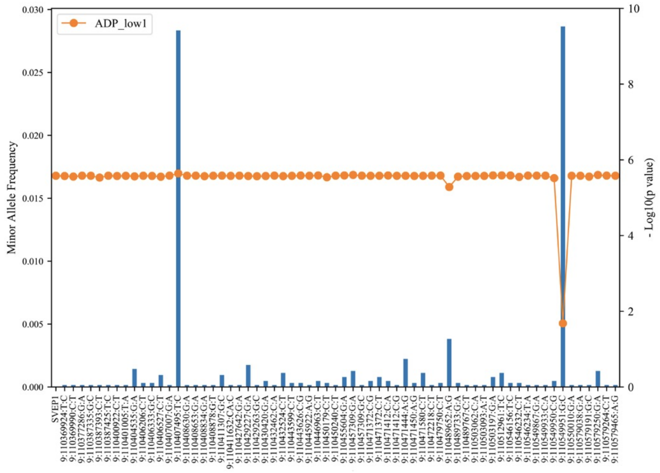

[B] BCO1,

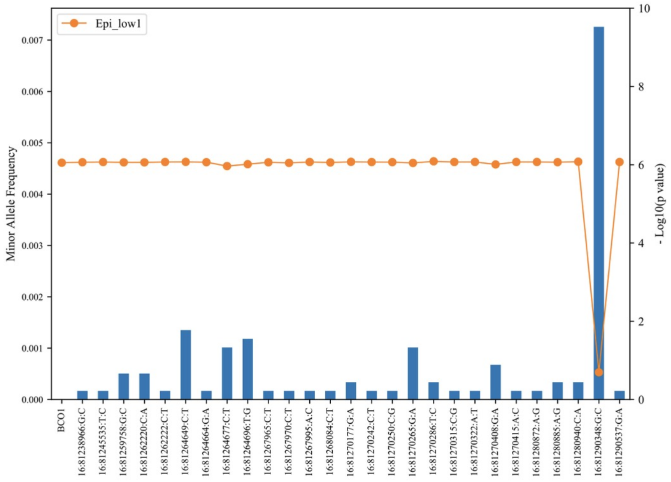

[C] NELFA

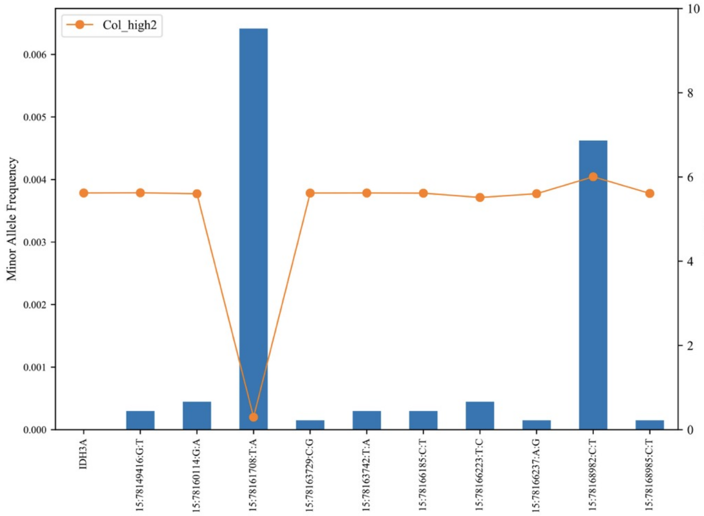

[D] IDH3A

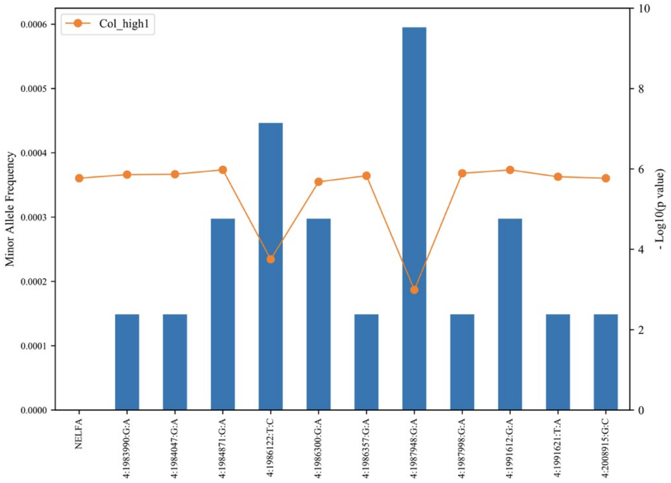

Supplementary Figure 8: QQ plots of SKAT analyses of rare non-coding variants in megakaryocyte specific super-enhancers.

Supplementary Figure 8: QQ plots of SKAT analyses of rare non-coding variants in megakaryocyte specific super-enhancers with ADP, Collagen and Epinephrine induced platelet aggregation. P-values are from a two-sided score test with no adjustment for multiple testing from the SKAT analysis.

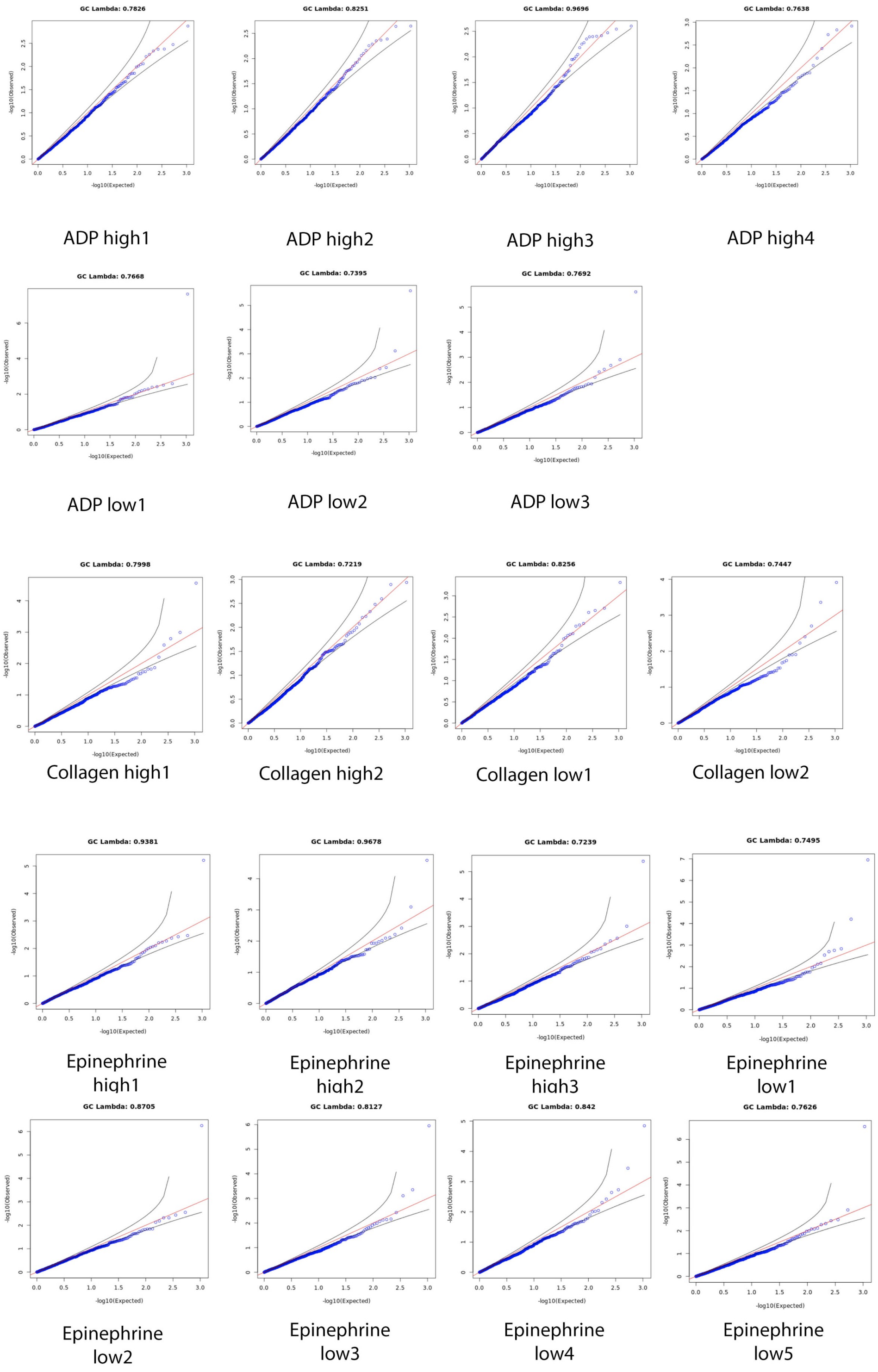

Supplementary Figure 9: Leave-one-out technique to identify the variants contributing the super enhancer located at *PEAR1* locus.

Supplementary Figure 9: Leave-one-out technique to identify the variants contributing the most to SKAT p-value of the super enhancer located at *PEAR1* locus. Blue dots represent the -Log10(p-value) of aggregated non-coding variants in *PEAR1* locus when the variant was left out. P-values are from two-sided score tests with no adjustment for multiple testing.

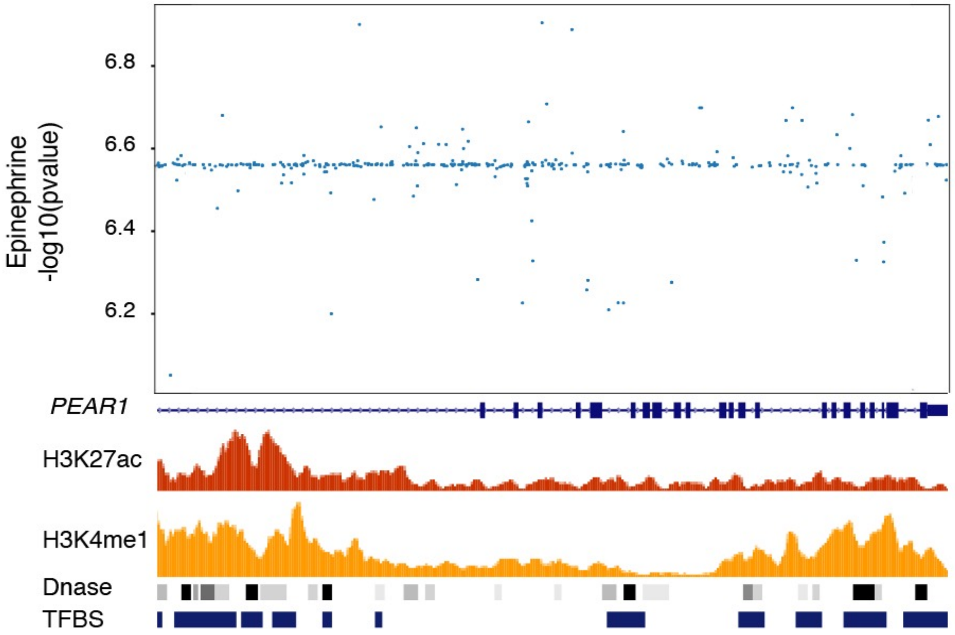

Supplement: Supplementary file 1 — Supplementary Information [file 41467_2021_23470_MOESM1_ESM.pdf]
