# Supplementary material for: Genome sequencing unveils a regulatory landscape of platelet reactivity
Source: Nat Commun. 2021 Jun 15;12:3626. doi: 10.1038/s41467-021-23470-9 (PMC8206369; doi:10.1038/s41467-021-23470-9)
Supplement: Supplementary file 3 — Description of Additional Supplementary Files [file 41467_2021_23470_MOESM3_ESM.pdf]

Description of Additional Supplementary Files

File Name: Supplementary Data 1
Description: Discovery and Replication results by 16 Discovered Loci and phenotypes. Replication was performed as a sample-size weighted meta-analysis of up to N=2,009 individuals in order of FHS, GeneSTAR European Americans, GeneSTAR African Americans and OOA for direction of effect. An imputation Rsq filter of 0.7 was applied in the meta-analysis. P-values are from a two-sided score test with no adjustment for discovery, and one sided Z test no adjustment for replication.

File Name: Supplementary Data 2
Description: The BioVU Biobank and UKBB PheWAS were queried for the top 16 independent variants from the single variant association signal (Table 1). The PheWAS query was limited to blood and cardiovascular traits as described in the methods. There were a total of 71 PheCodes, and each was looked up in African American (AA) and European American (EA) PheWAS from the BioVU Biobank, and UKBB as a whole. All PheCodes with a $p<0.05$ are listed in the table, and within each locus PheCodes are sorted by p-value. All ORs from the PheWAS are with respect to the 'effect' allele indicated the first column, and the effect size (Beta) from the significant platelet aggregation trait in Table 1 is also listed in column 1. P-values are from a two-sided score test with no adjustment for multiple testing.

File Name: Supplementary Data 3
Description: This is a list of the protein sequence identifier accessions for SVEP1 presented in the same order in which the sequences appear in the alignment in Figure 3C, along with the genus and species name and protein sequence length.
