# Supplementary material for: Genome sequencing unveils a regulatory landscape of platelet reactivity
Source: Nat Commun. 2021 Jun 15;12:3626. doi: 10.1038/s41467-021-23470-9 (PMC8206369; doi:10.1038/s41467-021-23470-9)
Supplement: Supplementary file 7 — Reporting Summary [file 41467_2021_23470_MOESM7_ESM.pdf]

nature research

Corresponding author(s): Joshua Lewis, Rasika Mathias, Andrew Johnson

Last updated by author(s): 3/24/2021

Reporting Summary

Nature Research wishes to improve the reproducibility of the work that we publish. This form provides structure for consistency and transparency in reporting. For further information on Nature Research policies, see Authors & Referees and the Editorial Policy Checklist .

Statistics

For all statistical analyses, confirm that the following items are present in the figure legend, table legend, main text, or Methods section.

n/a	Confirmed	
☐	☒	The exact sample size (*n*) for each experimental group/condition, given as a discrete number and unit of measurement
☐	☒	A statement on whether measurements were taken from distinct samples or whether the same sample was measured repeatedly
☐	☒	The statistical test(s) used AND whether they are one- or two-sided<br>*Only common tests should be described solely by name; describe more complex techniques in the Methods section.*
☐	☒	A description of all covariates tested
☐	☒	A description of any assumptions or corrections, such as tests of normality and adjustment for multiple comparisons
☐	☒	A full description of the statistical parameters including central tendency (e.g. means) or other basic estimates (e.g. regression coefficient) AND variation (e.g. standard deviation) or associated estimates of uncertainty (e.g. confidence intervals)
☐	☒	For null hypothesis testing, the test statistic (e.g. *F*, *t*, *r*) with confidence intervals, effect sizes, degrees of freedom and *P* value noted<br>*Give P values as exact values whenever suitable.*
☐	☒	For Bayesian analysis, information on the choice of priors and Markov chain Monte Carlo settings
☐	☒	For hierarchical and complex designs, identification of the appropriate level for tests and full reporting of outcomes
☐	☒	Estimates of effect sizes (e.g. Cohen's *d*, Pearson's *r*), indicating how they were calculated

Our web collection on statistics for biologists contains articles on many of the points above.

Software and code

Policy information about availability of computer code

Data collection	Sequencing QC and calling software GotCloud is described in a separate companion manuscript (Talian et al.) on the TOPMed WGS program. This published Nature manuscript is cited.
Data analysis	The following were additionally used for analyses in this manuscript: 1) WGSA v.7, 2) dbNSFP, 3) REVEL, 4) MCAP, 5) CADD, 6) Analysis Commons, 7) GENESIS (GENetic EStimation and Inference in Structure saamples), 8) SKAT, 9) METAL, 10) the University of Michigan Haplotype Reference Consortium imputation server, 11) EMMAX (Efficient Mixed-Model Assocation eXpedited), 12) MatrixEQTL, 13) HISAT2, 14) StringTie, 15) Ballgown, 16) the R statistical programming language (including packages for coloc and others), 17) the GeneATLAS webserver, 18) the UniProt protein BLAST server, 19) MAFFT v.7, 20) Minimac v.4, 21) Eagle, 22) Affymetrix Axiom analysis software

For manuscripts utilizing custom algorithms or software that are central to the research but not yet described in published literature, software must be made available to editors/reviewers. We strongly encourage code deposition in a community repository (e.g. GitHub). See the Nature Research guidelines for submitting code & software for further information.

Data

Policy information about availability of data

All manuscripts must include a data availability statement. This statement should provide the following information, where applicable:

- Accession codes, unique identifiers, or web links for publicly available datasets
- A list of figures that have associated raw data
- A description of any restrictions on data availability

A data availability statement has been added.

Field-specific reporting

Please select the one below that is the best fit for your research. If you are not sure, read the appropriate sections before making your selection.

- [x] Life sciences
- [ ] Behavioural & social sciences
- [ ] Ecological, evolutionary & environmental sciences

For a reference copy of the document with all sections, see nature.com/documents/nr-reporting-summary-flat.pdf

Life sciences study design

All studies must disclose on these points even when the disclosure is negative.

Sample size	Sample size was dependent on those cohort samples that attended exams and had platelet aggregation measurements conducted from fasting blood draws. Further, the sample was limited to those with WGS data and variant calling in the NHLBI TOPMed project (or imputation of genetic markers to the TOPMed reference panel along with phenotypes). Sample level exclusions were mainly for history of bleeding and cardiovascular disease, taking antiplatelet medications, and missing WGS or GWAS genetic data, or due to sex mismatch to genotype, pedigree errors or lack of concordance with prior GWAS genotyping data. These are described in further detail below and in the manuscript.
Data exclusions	Single variant analyses were limited to those samples with minor allele counts (MAC) of at least 5 and WGS sequencing depth of coverage of at least 10. For gene-based statistical tests aggregating multiple variants, we only included rare variants with minor allele frequency <5%, and for which there was an ENSEMBL deleterious annotation including the following: stop-gain, stop-loss, frameshift, or missense variants meeting one or more additional criteria -- REVEL score > 0.5, M_CAP score of "Deleterious", and/or CADD score >30. Variants in super-enhancer regulatory regions were limited to those with minor allele frequency <5%, and to those falling within megakaryocyte-specific super-enhancers as defined by Petersen et al. Nat. Commun. 8:16058 (2017).
Replication	Redundant statistical analyses were conducted be 2 analysts and compared to ensure consistency (and address any sources of discrepancy before final analyses). Independent population association replication was sought by utilizing SNP imputation into those samples with phenotypes that did not have WGS variant calling available, and in the independent Caerphilly GWAS sample imputed to HRC haplotype reference panel version 1.1
Randomization	All samples with available phenotypes and meeting inclusion criteria were included. There was no treatment or intervention. Thus, there was not randomization.
Blinding	This was an association study rather than case/control study or clinical trial so samples were not blinded.

Reporting for specific materials, systems and methods

We require information from authors about some types of materials, experimental systems and methods used in many studies. Here, indicate whether each material, system or method listed is relevant to your study. If you are not sure if a list item applies to your research, read the appropriate section before selecting a response.

Materials & experimental systems

n/a	Involved in the study	
x		Antibodies
x		Eukaryotic cell lines
x		Palaeontology
x		Animals and other organisms
	x	Human research participants
x		Clinical data

Methods

n/a	Involved in the study	
x		ChIP-seq
x		Flow cytometry
x		MRI-based neuroimaging

Human research participants

Policy information about studies involving human research participants

Population characteristics	This information is described in the manuscript text and tables. Briefly, the participants were drawn from 4 unique studies: the Framingham Heart Study (FHS) Offspring Cohort (exam 5), the Old Order Amish (OOA) PAPI study, the GeneSTAR European ancestry study (GS EA), and the GeneSTAR African ancestry (GS AA) study. The studies all had higher inclusion of females (FHS: 53%, OOA: 51%, GS EA: 56%, GS AA: 63%). All studies had the bulk of participants in middle age ranges (FHS: mean age 56 years, OOA: 47 years, GS EA: 45 years, GS AA: 43 years). Aspirin taking was an exclusion in GS EA, GS AA and OOA, and was discouraged in FHS (and tested for by standard arachidonic acid platelet light transmission aggregometry testing). Participants were excluded for cardiovascular disease history in GS EA and GS AA, for known bleeding disorders in GS EA, GS AA and OOA, for anticoagulant or antiplatelet medication (or unwillingness to discontinue) in GS EA, GS AA and OOA, as well as several other criteria. Overall the populations were relatively healthy individuals in middle age. The Caerphilly Prospective Study (CaPS) used in replication is an all-male study recruited from among the population in the rural Welsh town of Caerphilly, New South Wales. These individuals were also in middle age at the time of platelet measurement.
Recruitment	The FHS participants were originally enrolled at baseline as the children (and their spouses) of the FHS Original Cohort. The exam 5 FHS participants were recruited on the basis of having attended exam 4 and being in contact and willing to attend exam 5 and donate their blood. The GS EA and GS AA cohorts were recruited from apparently healthy family members of probands hospitalized for coronary disease among 10 Baltimore area hospitals. Thus, there may be some bias toward selection from families with a pre-existing history of cardiovascular disease. The Old Order Amish cohort is drawn from a unique founder population. Individuals were initially recruited at age >20 and were generally healthy at recruitment, and had to be willing to discontinue medications, supplements and vitamins for at least 1 week before testing.
Ethics oversight	Written informed consent was obtained for all studies and participants. The FHS study was approved by the Boston University Institutional Review Board. The GS EA and GS AA studies were approved by the Johns Hopkins Institutional Review Board. The OOA PAPI study was approved by the University of Maryland Institutional Review Board. Institutional approval for genotype-phenotype studies in the CaPs study was granted by the South East Wale Local Research Ethics Committee Panel B.

Note that full information on the approval of the study protocol must also be provided in the manuscript.
